# Implicit finite-difference schemes, based on the Rosenbrock method, for nonlinear Schrödinger equation with artificial boundary conditions

**DOI:** 10.1371/journal.pone.0206235

**Published:** 2018-10-31

**Authors:** Vyacheslav A. Trofimov, Evgeny M. Trykin

**Affiliations:** Department of Computational Methods, Faculty of Computational Mathematics and Cybernetics, Lomonosov Moscow State University, Moscow, Russian Federation; University of California Merced, UNITED STATES

## Abstract

We investigate the effectiveness of using the Rosenbrock method for numerical solution of 1D nonlinear Schrödinger equation (or the set of equations) with artificial boundary conditions (ABCs). We compare the computer simulation results obtained during long time interval at using the finite-difference scheme based on the Rosenbrock method and at using the conservative finite-difference scheme. We show, that the finite-difference scheme based on the Rosenbrock method is conditionally conservative one. To combine the advantages of both numerical methods, we propose new implicit and conditionally conservative combined method based on using both the conservative finite-difference scheme and conditionally conservative Rosenbrock method and investigate its effectiveness. The combined method allows decreasing the computer simulation time in comparison with the corresponding computer simulation time at using the Rosenbrock method. In practice, the combined method is effective at computation during short time interval, which does not require an asymptotic stability property for the finite-difference scheme. We generalize also the combined method with ABCs for 2D case.

## Introduction

As is well-known, wide physical phenomena (starting from laser radiation propagation in a nonlinear medium up to quantum mechanics problems and Bose-Einstein condensate (BEC)) are described by the nonlinear Schrödinger equation (or set of the equations). As a rule, its solution can be obtained based on the computer simulation using the finite-difference schemes. They can be conditionally divided on the three types. First, we should emphasize the conservative finite-difference schemes (CFDS) for the nonlinear Schrödinger equation. They were first proposed in [1], and then investigated, in detail, in papers [2–5] and other papers of these authors. These finite-difference schemes are nonlinear ones and, therefore, require an iterative process for their implementation, which leads to additional computation time costs. However, they possess the following evident advantages: the difference analogues of the problem invariants (conservation laws) are preserved. We keep in mind not only the energy conservation law (integral of motion) but also other invariants, in particular the Hamiltonian, which depends on the laser radiation phase. It is fundamental feature for the laser physics problem solution.

The second way of the finite-difference scheme construction for the problems under consideration is based on using the split-step method [6–12] (and many other papers), which artificially represent a nonlinear medium by alternating intervals with linear optical properties and with the nonlinear ones. This way does not allow us to construct the completely CFDS, which preserves all of the problem invariants. Usually, one can achieve only a preservation of the energy invariant difference analogue. However, using the iterative process on the stage of solving a nonlinear part of the Schrödinger equation [13–15], one can realize the conservation of the problem Hamiltonian with the first (or second) approximation order with respect to the spatial coordinate along which an optical pulse propagates. Another important disadvantage of this method is the asymptotic stability property absence. This property leads to the necessity of decreasing the spatial coordinate step significantly. The main evident advantage of the split-step method is the absence of an iterative process and the computation is carried out by explicit finite-difference schemes.

The third numerical method for the nonlinear Schrödinger equation solution can be conditionally related to the Rosenbrock method [16], which is widely used for the various physical and chemical problems solution [17–31]. As it well-known, it is explicit method, which is obtained by linearizing an implicit finite-difference scheme with a weight 0.5 (Crank-Nikolson scheme). As a result, the computation method becomes two-stages with real or complex parameters. For the nonlinear Schrödinger equation solution, the finite-difference scheme, based on the Rosenbrock method with real coefficient, is the most effective because the solution accuracy in this case is higher, in comparison with the Rosenbrock method, at using the complex coefficient. In particular, this result follows from the computer simulation provided both for the single nonlinear Schrödinger equation or for a set of these equations. The effectiveness of using the Rosenbrock method for the Schrödinger equation set solution is analyzed in this paper also because this case wasn’t widely discussed in the literature.

The main evident advantage of using the Rosenbrock method consists in its explicitness. However, this method is non-conservative one, to be more precise, it is conditionally conservative one with respect to some invariants (for example, to the problem Hamiltonian). This method does not also realize the asymptotic stability property of the finite-difference scheme.

Previously, a comparison of using the split-step method and the CFDS for the nonlinear Schrödinger equation solution or the set of these equations was performed in the literature in detail. This investigation is presented in particular in [14, 15] and it was demonstrated the advantages of the CFDS. In [9] it is shown the significant changing (about unity) of the third invariant (the Hamiltonian) of the problem at computation on the base of the split-step method. However, these papers do not contain the detailed comparison between the finite-difference scheme based on the Rosenbrock method and the CFDS, developed for the nonlinear Schrödinger equation. This gap is partially filled up below.

In the past decade, development of the artificial boundary conditions (ABCs) is the direction for increasing the computer simulation effectiveness. With respect to the Schrödinger equation the ABCs were proposed, for example, in [3–5] [32–39]. As a rule, these boundary conditions (BCs) were realized for the finite-difference schemes based on the split-step method in contrast to the CFDS used by us. Thus, the realization of the finite-difference schemes based on the Rosenbrock method in combination with the ABCs is of interest. The effectiveness of this method is investigated detailed below.

One more important topic of this paper is the combined method, developed on the base of both CFDS and Rosenbrock method (see also [40]), with the aim of adding the advantages of the methods under consideration. We propose this method for the most actual problem, namely: the nonlinear Schrödinger equation solution with the ABCs. It should be stressed, this problem didn’t consider in literature before.

This paper has the following structure. In paragraph 2 the nonlinear laser pulse propagation problem (or BEC evolution [41–51]) is stated for 1D case with zero-value BCs and the problem invariants (integrals of motions) are stated. We develop the finite-difference scheme based on the Rosenbrock method together with the Thomas algorithm for its solution. We show, that the finite-difference scheme, based on the Rosenbrock method with real coefficient equal 0.5, corresponds to using the half-sum of mesh solutions from the upper and low time layers at an approximation of the equation right part.

The main part of paragraph 2 is devoted to the computer simulation results. We analyze changing of the problem invariants in time as well as the convergence of the difference solution at decreasing grid steps. We compare also computer simulation time for using both finite-difference schemes.

The structure of the paragraph 3 coincides with the paragraph 2 structure. However, in this paragraph we consider the set of two nonlinear Schrödinger equations.

In the paragraph 4 we investigate the effectiveness of using the Rosenbrock method for a solution of the nonlinear Schrödinger equation with ABCs. As it is well-known, the statement of these BCs allows us to increase significantly the computer simulation effectiveness. For evaluating of the Rosenbrock method effectiveness for the problem solution with ABCs we compare the computer simulation results of this problem with the solution, obtained using the CFDS for the problem with zero-value BCs stated in big domain.

In paragraph 5 we propose the combined method, based on both methods: CFDS and Rosenbrock method. The effectiveness evaluation is made for the three cases of the optical pulse propagation: linear propagation and nonlinear propagation with positive and negative nonlinear coefficient.

In paragraph 6 we compare the effectiveness of using the CFDS, the Rosenbrock method and the combined method for a solution of the 2D nonlinear Schrödinger equation with ABCs. We demonstrate that the Rosenbrock method does not possess enough high effectiveness for the solution of multidimensional problem despite this method is explicit one. However, the combined method used together with original iterative process may be preferable in comparison with using the CFDS.

In a conclusion (paragraph 7) we formulate briefly the paper results.

## 1D Schrödinger equation

### Problem statement and problem invariants

As it is well known, the laser pulse propagation in a matter with cubic nonlinear response or its interaction with BEC in 1D case is described by the following dimensionless Schrödinger equation

$$\begin{array}{c} \frac{\partial A}{\partial t}+ıD_{z}\frac{\partial^{2}A}{\partial z^{2}}+ı\gamma VA+ı\psi {\left|A\right|}^{2}A=0,t>0,0<z<L_{z} \end{array}$$ (1)

with initial condition

$$\begin{array}{c} {A|}_{t=0}=e^{-{\left(z-L_{z_{c}}\right)}^{2}+ı\chi \left(z-L_{z_{c}}\right)}, \end{array}$$ (2)

corresponding to the pulse propagation in a positive direction of z-axis if the following inequality *D*_*z*_ *χ* > 0 is valid, and with the following BCs

$$\begin{array}{c} {A|}_{z=0,L_{z}}=0, \end{array}$$ (3)

for the finite function. In (1) *A*(*t*, *z*) is a complex amplitude; *t* is a time; *z* denotes a spatial coordinate; *L*_*z*_ is its maximal value; $L_{z_{c}}$ is a coordinate of the laser beam center at initial time moment; *V* = *V*(*z*) is a potential, which is $V=e^{-{\left(z-L_{v}\right)}^{m_{v}}}$, 0 ≤ *z* ≤ *L*_*z*_; *m*_*v*_ = 2; 6… for the BEC problem and is equal to 0 near the domain boundaries, *L*_*v*_ is a coordinate of the potential center. Let us note, that in case of the laser pulse propagation analysis, the function *V*(*z*) describe changing a dielectric permittivity along the medium. Parameters *D*_*z*_, *γ*, *ψ*, *χ* is the real coefficients.

With respect to the initial distribution of a complex amplitude we should emphasize two remarks. The first of them relates to using of a Gaussian beam and pulse in physical experiments dealing with laser physics because a laser generates as rule, such beams and phases. The second one is caused by existence of the linear Schrödinger equation solution for initial Gaussian distribution. Using this solution, we can compare numerical and analytical solutions of the equation and estimate an accuracy of various finite-difference schemes.

Let us note that often instead of the BCs (3) one requires that the complex amplitude and its derivatives in z-coordinate tends to zero at *z* → ± ∞:

$$\begin{array}{c} {A|}_{z\to \pm \infty }\to 0,\frac{\partial^{n}A}{\partial z^{n}}{|}_{z\to \pm \infty }\to 0,n\geq 1,n\in N. \end{array}$$ (4)

As we believe, for laser physics problem, the BCs (3) is preferable because the initial distribution of an electromagnetic field is always finite one and its propagation is analyzed during bounded interval of time. Therefore, below we will use the BCs (3). However, the problem invariants can be easy to generalize for unbounded domain in z-coordinate if the conditions (4) are valid.

The problem (1)–(3) possesses well-known invariants (the conservation laws):

$$\begin{array}{c} I_{1}\left(t\right)=\int_{0}^{L_{z}}{\left|A\right|}^{2}dz=const \end{array}$$ (5)

- the first invariant—energy invariant;

$$\begin{array}{c} I_{2}\left(t\right)=Im\int_{0}^{L_{z}}A^{*}\frac{\partial A}{\partial z}dz=const \end{array}$$ (6)

- the second invariant—impulse invariant (if *V*(*z*) = 0 and additional conditions $\frac{\partial A}{\partial z}{|}_{z=0,L_{z}}=0$ are valid);

$$\begin{array}{c} I_{3}\left(t\right)=\int_{0}^{L_{z}}(-D_{z}|\frac{\partial A}{\partial z}{|}^{2}+{\gamma V|A|}^{2}+\frac{\psi }{2}{\left|A\right|}^{4})dz=const \end{array}$$ (7)

- the third invariant—Hamiltonian. They are essential characteristics for controlling of the difference problem solution accuracy.

### Finite-difference scheme construction based on the Rosenbrock method

To develop a finite-difference scheme on the base of the Rosenbrock method [16] for the problem (1) we represent the complex amplitude by using the real and imaginary parts (note, that the modern computer can calculate in a complex arithmetic and this representation is not necessary for a method implementation)

$$\begin{array}{c} A(z,t)=u(z,t)+ıv(z,t). \end{array}$$ (8)

In the domain 0 ≤ *z* ≤ *L*_*z*_ we introduce a uniform grid:

$$\begin{array}{c} \omega_{z}=\left\{z_{j}=jh,j=0...N_{z},h=\frac{L_{z}}{N_{z}}\right\}. \end{array}$$ (9)

Let us define the grid functions in the grid nodes *ω*_*z*_

$$\begin{array}{c} {\bar{A}}_{j}={\bar{A}}_{h}\left(t,z_{j}\right)={\bar{u}}_{j}+ı{\bar{v}}_{j},{\bar{u}}_{j}={\bar{u}}_{h}\left(t,z_{j}\right),{\bar{v}}_{j}={\bar{v}}_{h}\left(t,z_{j}\right),V_{j}=V\left(z_{j}\right),0\leq j\leq N_{z}, \end{array}$$ (10)

and write the difference Laplace operator in the internal grid nodes

$$\begin{array}{c} \Lambda_{\bar{z}z}A_{j}=\frac{A_{j+1}-2A_{j}+A_{j-1}}{h^{2}},1\leq j\leq N_{z}-1. \end{array}$$ (11)

Since the Rosenbrock method was initially using for the ODE solution ([16, 18–29]) therefore, the problem (1) is reduced to the following form

$$\begin{array}{c} \frac{d\bar{U}\left(t\right)}{dt}=G\left(\bar{U}\right),\;j=\bar{1,N_{z}-1}, \end{array}$$ (12)

where $\bar{U}=({\bar{u}}_{0},{\bar{u}}_{1},...,{\bar{u}}_{N_{z}},{\bar{v}}_{0},{\bar{v}}_{1},...,{\bar{v}}_{N_{z}})$, and a vector $G(\bar{U})$ is defined as

$$G\left(\bar{U}\right)=\left(\begin{array}{c} G{\left(\bar{U}\right)}_{R}\\ G{\left(\bar{U}\right)}_{Im} \end{array}\right)=\left(\begin{array}{c} D_{z}\Lambda_{\bar{z}z}{\bar{v}}_{j}+\gamma V_{j}{\bar{v}}_{j}+\psi \left({\bar{u}}_{j}^{2}+{\bar{v}}_{j}^{2}\right){\bar{v}}_{j}\\ -D_{z}\Lambda_{\bar{z}z}{\bar{u}}_{j}-\gamma V_{j}{\bar{u}}_{j}-\psi \left({\bar{u}}_{j}^{2}+{\bar{v}}_{j}^{2}\right){\bar{u}}_{j} \end{array}\right)\begin{array}{c} ,j=\bar{1,N_{z}-1}. \end{array}$$ (13)

For zero-value BCs (3), the grid functions under consideration are equal to zero in the boundary grid nodes: ${\bar{u}}_{0}={\bar{u}}_{N_{z}}={\bar{v}}_{0}={\bar{v}}_{N_{z}}=0$. Therefore, the following equations

$$\begin{array}{c} \frac{d\bar{U}\left(t\right)}{dt}=0,\;j=0,N_{z} \end{array}$$ (14)

can be stated.

The next step in the finite-difference scheme constructing is a definition of the mesh function in time domain. For this purpose we introduce a uniform grid along the time coordinate in the domain 0 ≤ *t* ≤ *L*_*t*_:

$$\begin{array}{c} \omega_{t}=\left\{t_{m}=m\tau ,m=0...N_{t},\tau =\frac{L_{t}}{N_{t}}\right\}, \end{array}$$ (15)

and define on this mesh the following grid functions

$$\begin{array}{c} \begin{array}{c} A_{m,j}=A_{h}\left(t_{m},z_{j}\right)=u_{m,j}+ıv_{m,j},u_{m,j}=u_{h}\left(t_{m},z_{j}\right),\\ v_{m,j}=v_{h}\left(t_{m},z_{j}\right),0\leq j\leq N_{z},0\leq m\leq N_{t}, \end{array} \end{array}$$ (16)

and the vectors

$$\begin{array}{c} \begin{array}{cc} \hat{U}= & (u_{m+1,0},u_{m+1,1},\ldots ,u_{m+1,N_{z}},v_{m+1,0},v_{m+1,1},\ldots ,v_{m+1,N_{z}}),\\ U= & (u_{m,0},u_{m,1},\ldots ,u_{m,N_{z}},v_{m,0},v_{m,1},\ldots ,v_{m,N_{z}}). \end{array} \end{array}$$ (17)

Below for brevity, we omit the index h in a notation of the mesh functions.

According to the Rosenbrock method, the difference solution on the next time layer in the internal grid nodes belonging to *ω*_*z*_ mesh is computed as

$$\begin{array}{c} \hat{U}=U+\tau Re\;\bar{k},\;m=\bar{0,N_{t}-1} \end{array}$$ (18)

where $Re\bar{k}$ is a real part of the vector $\bar{k}$, which is a solution of the linear equations

$$\begin{array}{c} \begin{array}{cc} \left(E-\beta \tau G_{U}\right)\bar{k}=G\left(U\right), & j=\bar{1,N_{z}-1},\\ {\bar{k}}_{0}=0,{\bar{k}}_{N_{z}}=0, & m=\bar{0,N_{t}-1}. \end{array} \end{array}$$ (19)

The difference function *U* at zero node of the mesh in time domain is defined by the initial distribution of the complex amplitude.

Above a matrix *E* is the unity matrix, *G*_*U*_ is the Jacobian for the equations set (12), *G*(*U*) is the vector (13) with respect to the mesh function *U* (17). The parameter *β* is a complex one equal $\beta =\frac{1}{2}\left(1+ı\right)$ or the real one equal $\beta =\frac{1}{2}$. These values of the parameter *β* are examinated in [17–29]. The vector $\bar{k}$ has the following components

$$\begin{array}{c} \begin{array}{cc} \bar{k}= & (k_{u,0}+ı{\tilde{k}}_{u,0},k_{u,1}+ı{\tilde{k}}_{u,1},\ldots ,k_{u,N_{z}}+ı{\tilde{k}}_{u,N_{z}},\\ & k_{v,0}+ı{\tilde{k}}_{v,0},k_{v,1}+ı{\tilde{k}}_{v,1},\ldots ,k_{v,N_{z}}+ı{\tilde{k}}_{v,N_{z}}). \end{array} \end{array}$$ (20)

It should be stressed, that in our opinion the one-stage Rosenbrock scheme with parameter $\beta =\frac{1}{2}$ corresponds to the Eq (12) right part approximation by the half-sum of the mesh functions from the upper and current time layers (so called, the Crank-Nikolson scheme). This type of the approximation is preferable for the nonlinear Schrödinger equation with a cubic nonlinear response because it allows us to achieve the conservative property with respect to the Hamiltonian (the third invariant (7)). Therefore, we discuss this case in detail with the aim to represent the solution on the upper time layer (let’s denote this solution as $\hat{Y}$) in the following form

$$\begin{array}{c} \hat{Y}=Y+\frac{\tau }{2}(f\left(\hat{Y}\right)+f\left(Y\right)), \end{array}$$ (21)

where *Y* denotes the solution on the previous time layer. Then, let’s represent the function $f(\hat{Y})$ as the Taylor expansion

$$\begin{array}{c} f\left(\hat{Y}\right)=f\left(Y\right)+f_{Y}\left(Y\right)\left(\hat{Y}-Y\right) \end{array}$$ (22)

and substitute the Eqs (22) in (21). As a result, we obtain the following equation

$$\begin{array}{c} \hat{Y}=Y+\frac{\tau }{2}(2f\left(Y\right)+f_{Y}\left(Y\right)\left(\hat{Y}-Y\right)) \end{array}$$ (23)

and rewrite this equation in following form:

$$\begin{array}{c} \hat{Y}\left(1-\frac{\tau }{2}f_{Y}\left(Y\right)\right)=Y\left(1-\frac{\tau }{2}f_{Y}\left(Y\right)\right)+\tau f\left(Y\right), \end{array}$$ (24)

that means, that we obtain the Rosenbrock scheme with the parameter $\beta =\frac{1}{2}$:

$$\begin{array}{c} {}[E-\frac{\tau }{2}f_{Y}\left(Y\right)]\frac{\left(\hat{Y}-Y\right)}{\tau }=f\left(Y\right) \end{array}$$ (25)

(compare with the Eqs (18) and (19)). This construction of the Rosenbrock scheme and the representation (24) explains the conditional conservatism of the finite-difference scheme, based on the Rosenbrock method, for the nonlinear Schrödinger equation.

To solve the Eq (19), let’s represent the vector $\bar{k}$ and the coefficient *β* in the following way

$$\bar{k}=\left(\begin{array}{c} k_{u}\\ k_{v} \end{array}\right)+ı\left(\begin{array}{c} {\tilde{k}}_{u}\\ {\tilde{k}}_{v} \end{array}\right),\;\beta =\beta_{R}+ı\beta_{I}.$$ (26)

Then, the Eq (19) can be rewritten as

$$\begin{array}{ccc} \left(E-\beta_{R}\tau G_{U}\right)\left(\begin{array}{c} k_{u}\\ k_{v} \end{array}\right)+\beta_{I}\tau G_{U}\left(\begin{array}{c} {\tilde{k}}_{u}\\ {\tilde{k}}_{v} \end{array}\right) & = & \left(\begin{array}{c} G_{R}\left(U\right)\\ G_{I}\left(U\right) \end{array}\right),\\ -\beta_{I}\tau G_{U}\left(\begin{array}{c} k_{u}\\ k_{v} \end{array}\right)+\left(E-\beta_{R}\tau G_{U}\right)\left(\begin{array}{c} {\tilde{k}}_{u}\\ {\tilde{k}}_{v} \end{array}\right) & = & \left(\begin{array}{c} 0\\ 0 \end{array}\right),\;m=\bar{0,N_{t}-1}. \end{array}$$ (27)

Below, for clearness we write the Eq (27) in the component-wise form

$$\begin{array}{c} \begin{array}{c} \left(1-2\beta_{R}\tau \psi u_{j}v_{j}\right)k_{u_{j}}-\frac{\beta_{R}\tau D_{z}}{h^{2}}\left(k_{v_{j-1}}+k_{v_{j+1}}\right)+\tau \beta_{R}\left(\frac{2D_{z}}{h^{2}}-\gamma V_{j}-\psi u_{j}^{2}-3\psi v_{j}^{2}\right)k_{v_{j}}+\\ +2\beta_{I}\tau \psi u_{j}v_{j}{\tilde{k}}_{u_{j}}+\frac{\beta_{I}\tau D_{z}}{h^{2}}\left({\tilde{k}}_{v_{j-1}}+{\tilde{k}}_{v_{j+1}}\right)+\tau \beta_{I}\left(-\frac{2D_{z}}{h^{2}}+\gamma V_{j}+\psi u_{j}^{2}+3\psi v_{j}^{2}\right){\tilde{k}}_{v_{j}}=\\ =D_{z}\Lambda_{\bar{z}z}v+\gamma v_{j}V_{j}+\psi \left(u_{j}^{2}+v_{j}^{2}\right)v_{j},\\ \frac{\beta_{R}\tau D_{z}}{h^{2}}\left(k_{u_{j-1}}+k_{u_{j+1}}\right)+\tau \beta_{R}\left(-\frac{2D_{z}}{h^{2}}+\gamma V_{j}+\psi v_{j}^{2}+3\psi u_{j}^{2}\right)k_{u_{j}}+\left(1+2\beta_{R}\tau \psi u_{j}v_{j}\right)k_{v_{j}}-\\ -\frac{\beta_{I}\tau D_{z}}{h^{2}}\left({\tilde{k}}_{u_{j-1}}+{\tilde{k}}_{u_{j+1}}\right)+\tau \beta_{I}\left(\frac{2D_{z}}{h^{2}}-\gamma V_{j}-\psi v_{j}^{2}-3\psi u_{j}^{2}\right){\tilde{k}}_{u_{j}}-2\beta_{I}\tau \psi u_{j}v_{j}{\tilde{k}}_{v_{j}}=\\ =-D_{z}\Lambda_{\bar{z}z}u_{j}-\gamma u_{j}V_{j}-\psi \left(u_{j}^{2}+v_{j}^{2}\right)u_{j},\\ -2\beta_{I}\tau \psi u_{j}v_{j}k_{u_{j}}-\frac{\beta_{I}\tau D_{z}}{h^{2}}\left(k_{v_{j-1}}+k_{v_{j+1}}\right)+\tau \beta_{I}\left(\frac{2D_{z}}{h^{2}}-\gamma V_{j}-\psi u_{j}^{2}-3\psi v_{j}^{2}\right)k_{v_{j}}+(1-\\ -2\beta_{R}\tau \psi u_{j}v_{j}){\tilde{k}}_{u_{j}}-\frac{\beta_{R}\tau D_{z}}{h^{2}}\left({\tilde{k}}_{v_{j-1}}+{\tilde{k}}_{v_{j+1}}\right)+\tau \beta_{R}\left(\frac{2D_{z}}{h^{2}}-\gamma V_{j}-\psi u_{j}^{2}-3\psi v_{j}^{2}\right){\tilde{k}}_{v_{j}}=0,\\ \frac{\beta_{I}\tau D_{z}}{h^{2}}\left(k_{u_{j-1}}+k_{u_{j+1}}\right)+\tau \beta_{I}\left(\gamma V_{j}-\frac{2D_{z}}{h^{2}}+\psi v_{j}^{2}+3\psi u_{j}^{2}\right)k_{u_{j}}+2\beta_{I}\tau \psi u_{j}v_{j}k_{v_{j}}+\\ +\frac{\beta_{R}\tau D_{z}}{h^{2}}\left({\tilde{k}}_{u_{j-1}}+{\tilde{k}}_{u_{j+1}}\right)+\tau \beta_{R}\left(-\frac{2D_{z}}{h^{2}}+\gamma V_{j}+\psi v_{j}^{2}+3\psi u_{j}^{2}\right){\tilde{k}}_{u_{j}}+\\ +\left(1+2\beta_{R}\tau \psi u_{j}v_{j}\right){\tilde{k}}_{v_{j}}=0,\;j=\bar{1,N_{z}-1},m=\bar{0,N_{t}-1}. \end{array} \end{array}$$ (28)

which can be also rewrite in the matrix form

$$\begin{array}{c} -Q{\hat{Y}}_{j-1}+C_{j}{\hat{Y}}_{j}-B{\hat{Y}}_{j+1}=F_{j}\left(U_{j}\right),\;{\hat{Y}}_{j}={\left(k_{u_{j}},k_{v_{j}},{\tilde{k}}_{u_{j}},{\tilde{k}}_{v_{j}}\right)}^{T},1\leq j\leq N_{z}-1. \end{array}$$ (29)

In (29) the matrices *Q*, *B*, *C*_*j*_ are defined as

$$\begin{array}{c} C_{j}=(\begin{array}{cccc} c_{1} & c_{2} & c_{3} & c_{4}\\ c_{5} & c_{6} & c_{7} & c_{8}\\ c_{9} & c_{10} & c_{11} & c_{12}\\ c_{13} & c_{14} & c_{15} & c_{16} \end{array}), \end{array}$$

$$\begin{array}{c} c_{1}=c_{11}=1-2\beta_{R}\tau \psi u_{j}v_{j};\;c_{5}=c_{15}=\tau \beta_{R}\left(-\frac{2D_{z}}{h^{2}}+\gamma V_{j}+\psi v_{j}^{2}+3\psi u_{j}^{2}\right);\\ c_{2}=c_{12}=\tau \beta_{R}\left(\frac{2D_{z}}{h^{2}}-\gamma V_{j}-\psi u_{j}^{2}-3\psi v_{j}^{2}\right);\;c_{6}=c_{16}=1+2\beta_{R}\tau \psi u_{j}v_{j};\\ c_{3}=-c_{8}=-c_{9}=c_{13}=2\beta_{I}\tau \psi u_{j}v_{j};\;c_{7}=\tau \beta_{I}\left(\frac{2D_{z}}{h^{2}}-\gamma V_{j}-\psi v_{j}^{2}-3\psi u_{j}^{2}\right);\\ c_{4}=-c_{10}=c_{14}=\tau \beta_{I}\left(\gamma V_{j}-\frac{2D_{z}}{h^{2}}+\psi u_{j}^{2}+3\psi v_{j}^{2}\right),j=\bar{1,N_{z}-1},m=\bar{0,N_{t}-1}, \end{array}$$

$$\begin{array}{c} Q=B=(\begin{array}{cccc} 0 & \frac{\beta_{R}\tau D_{z}}{h^{2}} & 0 & -\frac{\beta_{I}\tau D_{z}}{h^{2}}\\ -\frac{\beta_{R}\tau D_{z}}{h^{2}} & 0 & \frac{\beta_{I}\tau D_{z}}{h^{2}} & 0\\ 0 & \frac{\beta_{I}\tau D_{z}}{h^{2}} & 0 & \frac{\beta_{R}\tau D_{z}}{h^{2}}\\ -\frac{\beta_{I}\tau D_{z}}{h^{2}} & 0 & -\frac{\beta_{R}\tau D_{z}}{h^{2}} & 0 \end{array}). \end{array}$$

The right parts of Eq (29) are written in the following form

$$\begin{array}{c} \begin{array}{c} F_{j}\left(U_{j}\right)={\left({G{\left(U\right)}_{R}}_{j},{G{\left(U\right)}_{I}}_{j},0,0\right)}^{T},\;{G{\left(U\right)}_{R}}_{j}=D_{z}\Lambda_{\bar{z}z}v_{j}+\gamma V_{j}v_{j}+\psi \left(u_{j}^{2}+v_{j}^{2}\right)v_{j},\\ {G{\left(U\right)}_{I}}_{j}=-D_{z}\Lambda_{\bar{z}z}u_{j}-\gamma V_{j}u_{j}-\psi \left(u_{j}^{2}+v_{j}^{2}\right)u_{j},\;j=\bar{1,N_{z}-1},m=\bar{0,N_{t}-1}. \end{array} \end{array}$$ (30)

An effective method for solving the Eq (29) is the Thomas algorithm. According to this method, the solution can be found in following way

$$\begin{array}{c} {\hat{Y}}_{j}=\alpha_{j+1}{\hat{Y}}_{j+1}+\varsigma_{j+1},\;j=N_{z}-1,...,1,\;{\hat{Y}}_{0}=0,\;{\hat{Y}}_{N_{z}}=0, \end{array}$$ (31)

where *α*_*j*_ is a matrix of dimension (4x4); *ς_j_* is a vector of dimension 4, which are computed as follows

$$\begin{array}{c} \begin{array}{cccc} \alpha_{j+1}= & {\left(C_{j}-Q\alpha_{j}\right)}^{-1}B, & j=1,...,N_{z}-1; & \alpha_{1}={C_{1}}^{-1}B,\\ \varsigma_{j+1}= & {\left(C_{j}-Q\alpha_{j}\right)}^{-1}\left(F_{j}+Q\varsigma_{j}\right), & j=1,...,N_{z}-1; & \varsigma_{1}={C_{1}}^{-1}F_{1}. \end{array} \end{array}$$ (32)

The finite-difference scheme based on the Rosenbrock method possesses the second order of approximation on the spatial coordinate and the first order of approximation on the time coordinate. This scheme possesses also a property of the conditional conservatism, as it will be shown below.

### Conservative finite-difference scheme

Let us use the same grids, which are written above, and define the mesh functions:

$$\begin{array}{c} \begin{array}{c} A=A_{j}=A_{m,j}=A\left(t_{m},z_{j}\right),\;\hat{A}={\hat{A}}_{j}={\hat{A}}_{m+1,j}=A\left(t_{m}+\tau ,z_{j}\right),\\ \overset{0.5}{A}=0.5\left(\hat{A}+A\right),\;|\overset{0.5}{A}{|}^{2}=0.5(|\hat{A}{|}^{2}+{\left|A\right|}^{2}). \end{array} \end{array}$$ (33)

Then, let’s write following two-layers scheme for the Eq (1):

$$\begin{array}{c} \frac{\hat{A}-A}{\tau }+ıD_{z}\Lambda_{\bar{z}z}\overset{0.5}{A}+ıV\overset{0.5}{A}+ı\psi {\left|\overset{0.5}{A}\right|}^{2}\overset{0.5}{A}=0,\;j=\bar{1,N_{z}-1},m=\bar{0,N_{t}-1} \end{array}$$ (34)

with initial condition

$$\begin{array}{c} {A_{0}}_{j}=A_{0}\left(z_{j}\right),j=\bar{0,N_{z}} \end{array}$$ (35)

and BCs:

$$\begin{array}{c} {\hat{A}}_{0}={\hat{A}}_{N_{z}}=0. \end{array}$$ (36)

The finite-difference scheme (34–36) is nonlinear one, that is why for its solution we use an iteration process in following way:

$$\begin{array}{c} \frac{\overset{s+1}{\hat{A}}-A}{\tau }+ıD_{z}\Lambda_{\bar{z}z}\overset{s+1}{\overset{0.5}{A}}+ıV\overset{s+1}{\overset{0.5}{A}}+ı\psi {\left|\overset{s}{\overset{0.5}{A}}\right|}^{2}\overset{s}{\overset{0.5}{A}}=0,\;j=\bar{1,N_{z}-1},m=\bar{0,N_{t}-1}. \end{array}$$ (37)

The BCs for the equations are:

$$\begin{array}{c} \overset{s+1}{{\hat{A}}_{0}}=\overset{s+1}{{\hat{A}}_{N_{z}}}=0. \end{array}$$ (38)

The mesh function on the upper time layer at zero iteration (*s* = 0) is chosen as

$$\begin{array}{c} \overset{s=0}{\hat{A}}=A. \end{array}$$ (39)

The iteration process is stopped if the following condition, for example, is valid

$$\begin{array}{c} \underset{z_{j}}{max}|\overset{s+1}{\hat{A}}-\overset{s}{\hat{A}}|\leq {\tilde{\theta }}_{1}\underset{z_{j}}{max}\left|\overset{s}{\hat{A}}\right|+{\tilde{\theta }}_{2}, \end{array}$$ (40)

where ${\tilde{\theta }}_{1},{\tilde{\theta }}_{2}>0$ are the real constants.

Let us rewrite the Eq (37) in the matrix form:

$$\begin{array}{c} \begin{array}{c} -\bar{Q}\overset{s+1}{{\hat{A}}_{j+1}}+\bar{C}\overset{s+1}{{\hat{A}}_{j}}-\bar{B}\overset{s+1}{{\hat{A}}_{j-1}}={\bar{F}}_{j}\left(A_{j},A_{j-1},A_{j+1},\overset{s}{{\hat{A}}_{j}}\right),\\ A_{j}=(\begin{array}{c} u_{j}\\ v_{j} \end{array}),\overset{s+1}{{\hat{A}}_{j}}=(\begin{array}{c} \overset{s+1}{{\hat{u}}_{j}}\\ \overset{s+1}{{\hat{v}}_{j}} \end{array}),\bar{Q}=\bar{B}=(\begin{array}{cc} 0 & \tau D_{z}/2h^{2}\\ -\tau D_{z}/2h^{2} & 0 \end{array}),\\ \bar{C}=(\begin{array}{cc} 1 & -(0.5V\tau +\tau D_{z}/h^{2})\\ 0.5V\tau +\tau D_{z}/h^{2} & 1 \end{array}), \end{array} \end{array}$$ (41)

${\bar{F}}_{j}$ is a vector of dimension 2, which has the form:

$$\begin{array}{c} \begin{array}{c} {\bar{F}}_{j}={\bar{F}}_{j}\left(A_{j},A_{j-1},A_{j+1},\overset{s}{{\hat{A}}_{j}}\right)=-{\bar{Q}}^{\tau }A_{j+1}+{\bar{C}}^{\tau }A_{j}-{\bar{B}}^{\tau }A_{j-1}+\hat{F}\left(\overset{s}{\hat{A}}\right),\\ \hat{F}\left(\overset{s}{\hat{A}}\right)=(\begin{array}{c} \frac{\psi \tau (|\overset{s}{{\hat{A}}_{j}}{|}^{2}+|A_{j}{|}^{2})\left(\overset{s}{{\hat{v}}_{j}}+v_{j}\right)}{4}\\ -\frac{\psi \tau (|\overset{s}{{\hat{A}}_{j}}{|}^{2}+|A_{j}{|}^{2})\left(\overset{s}{{\hat{u}}_{j}}+u_{j}\right)}{4} \end{array}). \end{array} \end{array}$$ (42)

To solve this equations set we use the Thomas algorithm also. According to this method, the Eq (41) solution can be written in following way

$$\begin{array}{c} \overset{s+1}{{\hat{A}}_{j}}=\sigma_{j+1}\overset{s+1}{{\hat{A}}_{j+1}}+\varphi_{j+1},\;j=\bar{N_{z}-1,0}, \end{array}$$ (43)

where *σ* and *φ* are computed in following way:

$$\begin{array}{c} \begin{array}{cccc} \sigma_{j+1}= & {\left(\bar{C}-\bar{Q}\sigma_{j}\right)}^{-1}\bar{B}, & j=1,...,N_{z}-1; & \sigma_{1}={{\bar{C}}_{1}}^{-1}\bar{B},\\ \varphi_{j+1}= & {\left(\bar{C}-\bar{Q}\sigma_{j}\right)}^{-1}\left({\bar{F}}_{j}+\bar{Q}\varphi_{j}\right), & j=1,...,N_{z}-1; & \varphi_{1}={{\bar{C}}_{1}}^{-1}{\bar{F}}_{1}. \end{array} \end{array}$$ (44)

### The computer simulation results

#### Conservatism investigation of the finite-difference scheme based on the Rosenbrock method

For brevity, we define following notations: index *Ros*_1_ denotes a finite-difference scheme based on the Rosenbrock method with parameter *β* = 0.5 + 0.5*ı*; index *Ros*_2_ denotes the finite-difference scheme based on the Rosenbrock method with parameter *β* = 0.5. Index *C* denotes the CFDS. Correspondingly, the results obtained using considered finite-difference schemes we define as $A_{Ros_{1}}\left(t_{m},z_{j}\right),A_{Ros_{2}}\left(t_{m},z_{j}\right),A_{C}\left(t_{m},z_{j}\right)$. We compare these results with the aim to demonstrate the efficiency of using the Rosenbrock method. In particular, we analyze the difference between the grid functions:

$$\begin{array}{c} \begin{array}{cc} \delta A_{12}=\delta A_{Ros_{1}Ros_{2}}= & \max_{1\leq j\leq N_{z}}\left|\right|A_{Ros_{1}}\left(t_{m},z_{j}\right){|}^{2}-|A_{Ros_{2}}\left(t_{m},z_{j}\right){|}^{2}|,\\ \delta A_{C1}=\delta A_{CRos_{1}}= & \max_{1\leq j\leq N_{z}}\left|\right|A_{Ros_{1}}\left(t_{m},z_{j}\right){|}^{2}-|A_{C}t_{m},z_{j}{)|}^{2}|,\\ \delta A_{C2}=\delta A_{CRos_{2}}= & \max_{1\leq j\leq N_{z}}\left|\right|A_{Ros_{2}}\left(t_{m},z_{j}\right){|}^{2}-|A_{C}\left(t_{m},z_{j}\right){|}^{2}|. \end{array} \end{array}$$ (45)

For the effectiveness estimation of the finite-difference schemes we analyze changing the invariants between their values at time moment *t* = *L*_*t*_ and at initial time moment:

$$\begin{array}{c} \delta I_{1}=|I_{1}\left(L_{t}\right)-I_{1}\left(0\right)|,\;\delta I_{3}=\left|I_{3}\left(L_{t}\right)-I_{3}\left(0\right)\right|. \end{array}$$ (46)

Let us define also the maximal intensity on the time layer *m* in the following way:

$$\begin{array}{c} {\left|A\right|}_{max}^{2}=\max_{1\leq j\leq N_{z}}|A_{j}{|}^{2}. \end{array}$$ (47)

The parameters *D*_*z*_, *χ* at the computer simulation is equal to *D*_*z*_ = 1.0, *χ* = 0 (the pulse does not move). As a rule, the evolution of the solution is analyzed during 10 dimensionless units (*L*_*t*_ = 10.0) if it’s not emphasized and maximal value of the spatial coordinate is equal to 40 dimensionless units (*L*_*z*_ = 40.0). The initial distributions center placed in section $L_{z_{c}}=20.0$ It allows us to analyze also the asymptotic stability property of finite-difference scheme because this time interval is big enough for the problem under consideration with chosen parameters. In the numerical simulation we use also the following values of the iteration parameters ${\tilde{\theta }}_{1}=10^{-3},{\tilde{\theta }}_{2}=10^{-5}$ and we choose the parameters of the potential as *L*_*v*_ = 20.0, *m*_*v*_ = 2.

**Fig 1:**
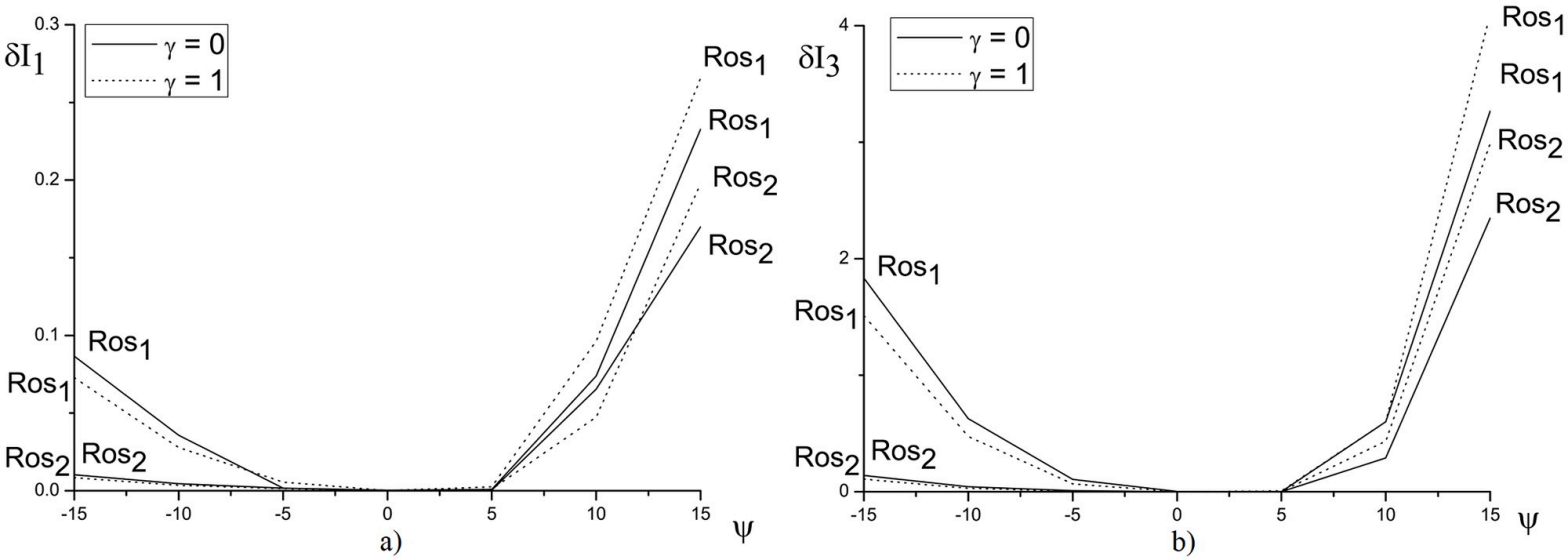
The dependence of *δI*_1_(*a*) and *δI*_3_(*b*) from the nonlinear coefficient *ψ* for the parameters *γ* = 0 and 1, and the mesh steps *τ* = *h* = 0.01.

In Fig 1 the invariant deviation in depending on the nonlinear coefficient is shown. We see that with increasing of the nonlinearity coefficient, the inaccuracy for the first and third invariants computation increases. The greatest invariants deviation is observed for the positive value of the parameter *ψ*, that corresponds to the optical pulse self-focusing.

**Fig 2:**
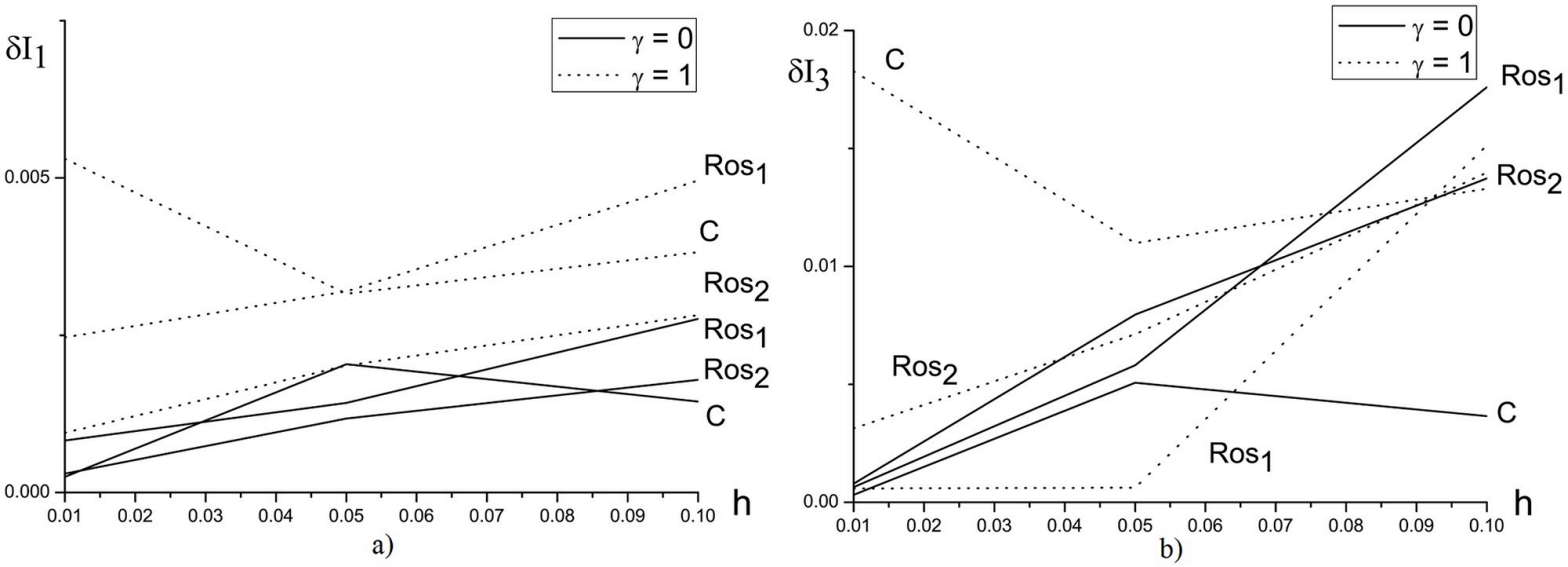
The dependence of *δI*_1_(*a*) and *δI*_3_(*b*) from the mesh step *h* for the parameter *ψ* = 5.0 and the mesh step *τ* = 0.01 at the time moment *t* = 10.

**Fig 3:**
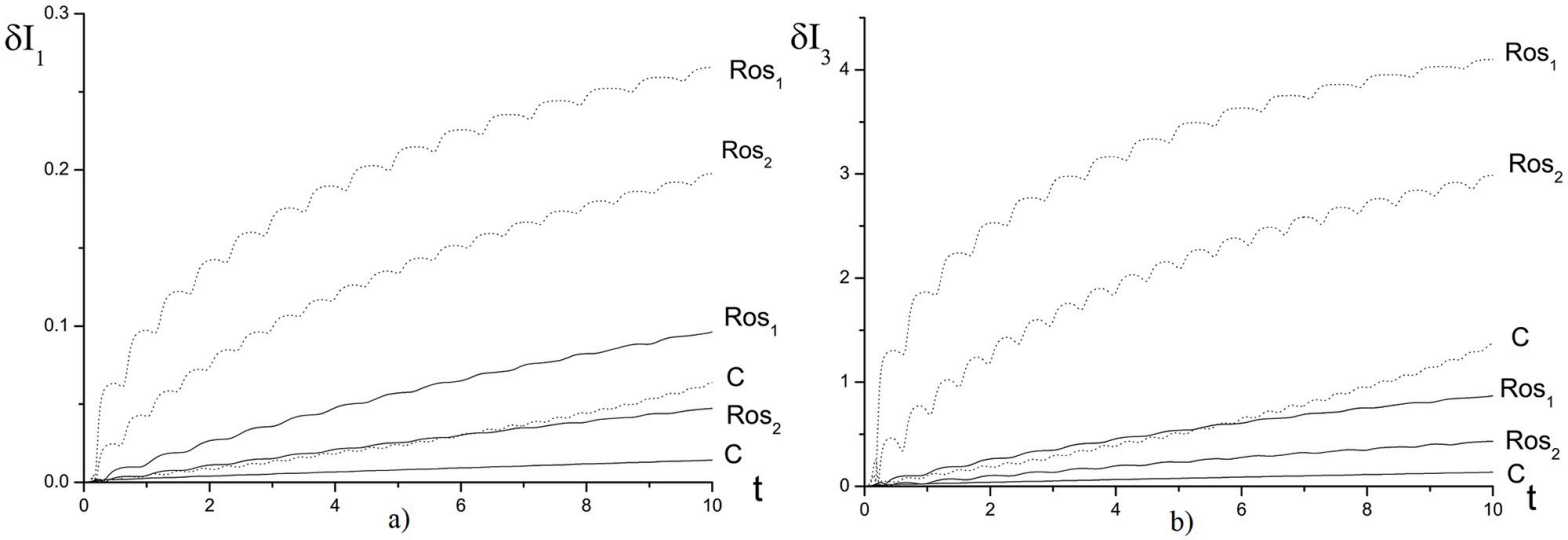
The evolution of the first *δI*_1_ (a) and the third *δI*_3_ (b) invariant deviation in time, computed using various methods for the parameters *ψ* = 10.0 (solid line), 15.0 (dotted line), *γ* = 1.0, *h* = *τ* = 0.01.

Fig 2 demonstrates the invariant deviation depending on the mesh step *h* in time moment *t* = 10. We emphasize, that the similar results obtained using the finite-difference schemes under consideration for the various parameter values. We see that the Hamiltonian is conserved by the Rosenbrock method with the complex coefficient *β* for *h* ≤ 0.05 at computer simulation of the laser pulse propagation in a medium with self-focusing nonlinear response and potential equal unity. However, with increasing the nonlinear coefficient and duration of a considered time interval, the invariant deviation for the solution, obtained using Rosenbrock method, increases faster in comparison with the corresponding values, obtained using the CFDS (Fig 3).

The invariants changing also occurs for the computation results obtained on the base of the CFDS for time step *τ* = 0.01. It is due to chosen iteration parameters. The Hamiltonian changing depicted in Fig 2b, in our opinion, can be explained by the finite-difference scheme spectral properties: with increasing the mesh step on z-coordinate a number of the difference solution spectral components decreases.

**Fig 4:**
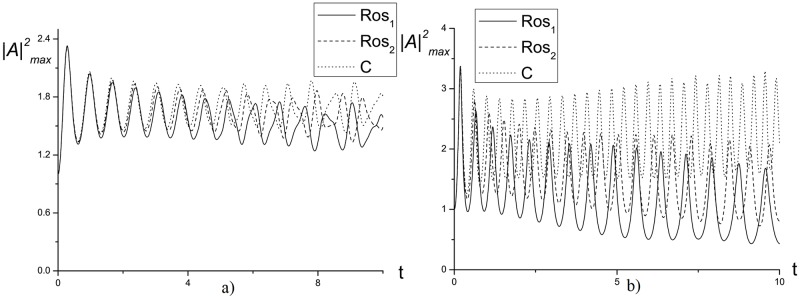
The evolution of the maximal intensity ${\left|A\right|}_{max}^{2}$ in time, computed using various methods for the parameters *ψ* = 10.0(*a*), 15.0(*b*), *γ* = 1.0, *h* = *τ* = 0.01.

For practice, it is of interest the first invariant and Hamiltonian evolution in time, which is presented in Figs 3 and 4, because they define the problem solution and also the maximal solution intensity. Note, that in Fig 3 the invariants changing increases with the time. However, the finite-difference scheme *Ros*_2_ has the lower deviation in comparison with the finite-difference scheme *Ros*_1_. Non-conservation of the invariants affects to the laser pulse maximal intensity evolution, which is shown in Fig 4. As it is well-seen, it has the oscillating character for the process (*γ* = 1). The Schrödinger equation solution, obtained using the CFDS, saves the oscillation period in contrast to the solution, obtained using the finite-difference scheme based on the Rosenbrock method. In the last case, the increasing oscillation period at growing the computation time takes place. Moreover, it should be stressed, that for the solution, obtained using the CFDS, the maximal intensity deviation is substantially less and it corresponds to the iterative process accuracy. Therefore, the Rosenbrock method accumulates the computation errors in time.

**Fig 5:**
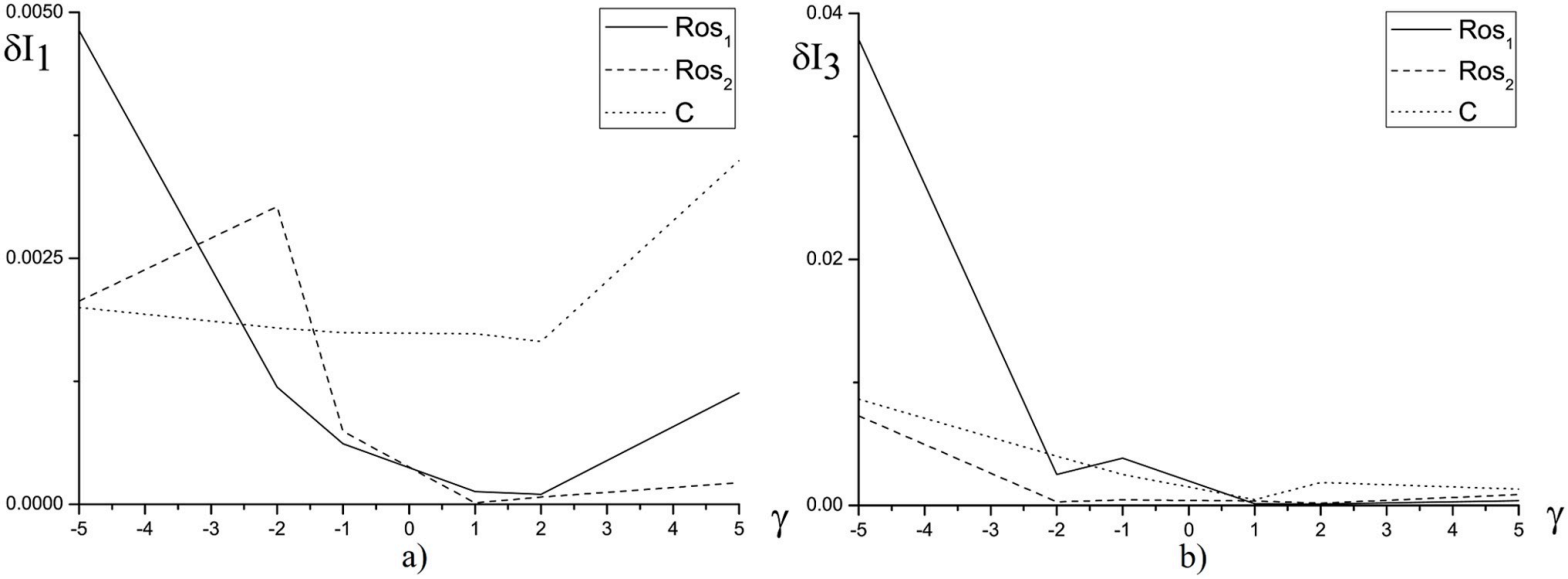
The invariant deviation *δI*_1_(*a*) and *δI*_3_(*b*) in dependence of the parameter *γ* at time moment *t* = 10 and parameter *ψ* = 1.0 and for the mesh steps *h* = *τ* = 0.01.

**Fig 6:**
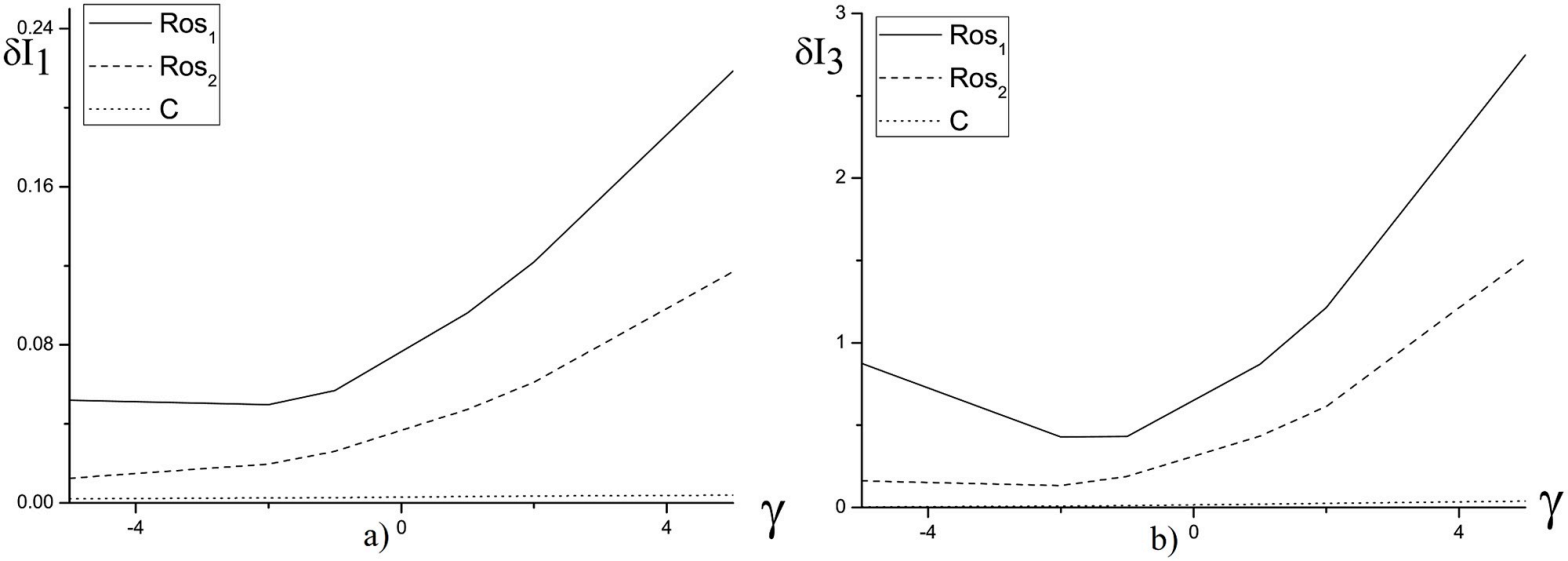
The invariant deviation *δI*_1_(*a*) and *δI*_3_(*b*) in dependence of the parameter *γ* for the fixed parameters *ψ* = 10.0, *L*_*t*_ = 10.0, *L*_*z*_ = 40.0 and the mesh steps *h* = *τ* = 0.01.

Let us consider the influence of the external potential on the problem invariant violations at other fixed parameters: *L*_*t*_ = 10.0, *h* = *τ* = 0.01. This dependencies are shown in Figs 5 and 6 and in Table 1 for various values of the nonlinear coefficient (*ψ*). We see in Fig 5 that the first and third invariants errors grow with increasing of the parameter |*γ*| and the maximal deviation occurs for the negative parameter *γ*. At the weak nonlinearity (*ψ* = 1) and positive parameter *γ*, the invariants deviations at using the finite-difference schemes based on Rosenbrock method is less than the corresponding values at using the CFDS. This strange feature causes by chosen iteration process parameters.

**Table 1:** The first *I*_1_ and third *I*_3_ invariant deviation, computed for the mesh steps *τ* = 0.05 and *h* = 0.01 at the time moment *t* = 10 for the parameter *ψ* = 5.0.

		*Ros*_1_	*Ros*_2_	*C*
*δI*_1_	*γ* = 0	0.0683993	0.0303243	0.0109565
	*γ* = 1	0.1785331	0.0875587	0.0150573
*δI*_3_	*γ* = 0	0.1313467	0.0707009	0.0263863
	*γ* = 1	0.5269990	0.2849560	0.0527133

With increasing the nonlinear coefficient up to *ψ* = 5.0 (Table 1) or *ψ* = 10.0 (Fig 6) the Rosenbrock schemes preserve the invariants essentially worse. So, Table 1 demonstrates that at *ψ* = 5.0 and the external potential absence, the third invariant *I*_3_ preserves 6 times worse at using the finite-difference scheme *Ros*_1_ and 3 times worse at using the finite-difference scheme *Ros*_2_ in comparison with computation on the base of the CFDS. If the external potential is preset then the Hamiltonian changes even more: the error of invariant computation increases to 10 or 5 times if a computer simulation is provided on the base of the schemes *Ros*_1_ and *Ros*_2_, correspondingly. The first invariant changing for the Rosenbrock schemes also becomes unacceptable. Further increasing of the parameter *ψ* (see Fig 6) results in tens times enhancement of the invariant errors at using the finite-difference schemes *Ros*_1_ and *Ros*_2_. However, the corresponding invariant deviation is insignificant at using the CFDS. This fact is a consequence of the Rosenbrock method conditional conservatism if the optical pulse propagation in a nonlinear medium is analyzed. Therefore, with decreasing the time step, the invariant deviations for numerical solutions, obtained using the Rosenbrock methods, tend to the corresponding values, obtained using the CFDS. We demonstrate this feature in Table 2: for the time step *τ* = 0.001 the first invariant and Hamiltonian deviations occurring at using the Rosenbrock methods and the CFDS have the similar order of magnitude and numerical solutions for all methods coincide each other. However, we stress that the numerical solution of the problem possesses enough high accuracy if a computation is provided on the base of the CFDS with time step *τ* = 0.01.

**Table 2:** The invariant deviation for parameters *ψ* = 20.0, *γ* = −1.0 and mesh step *h* = 0.01 depending on the time step.

Scheme	*δI*_1_(*τ* = 0.01)	*δI*_3_(*τ* = 0.01)	*δI*_1_(*τ* = 0.001)	*δI*_3_(*τ* = 0.001)
*Ros*_1_	0.152964	4.592049	0.00039	0.012824
*Ros*_2_	0.1067	2.523858	0.000147	0.002358
*C*	0.000933	0.010421	0.000831	0.0028492

**Fig 7:**
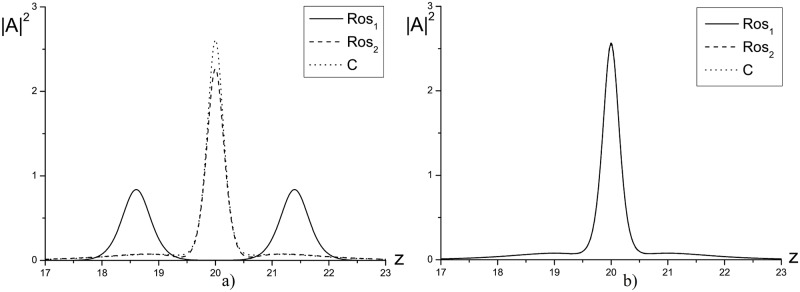
The intensity profile |*A*|^2^, depicted for the coordinate interval 17.0 ≤ *z* ≤ 23.0 at the time moment *t* = 1.0 and computed for the parameters *ψ* = 20.0, *γ* = −1.0 and the time steps *τ* = 0.01(*a*), 0.001(*b*).

Thus, at the fixed mesh step *h* and fixed time interval we can chose the time step *τ* for the Rosenbrock method in such a way, that the invariants preserve with sufficient high accuracy and the numerical solution obtained using this method tends to the corresponding solution, obtained using the CFDS. This statement is confirmed, in particular, in Fig 7 and Table 3. Essentially, that the solutions, obtained using the CFDS for the time steps *τ* = 0.01 and *τ* = 0.001, are practically identical each other. So, for this scheme can obtain the accurate result of a computation at using the mesh step on time coordinate equal *τ* = 0.01, which allows to decrease significantly the computation time. Therefore, the computation efficiency is more high for the CFDS.

**Table 3:** The maximal solution difference, obtained using the finite-difference scheme based on the Rosenbrock method and the CFDS.

*τ*	*δA*_12_	*δA*_*C*1_	*δA*_*C*2_
0.01	2.268552552	2.610536552	0.341984
0.001	0.011948	0.011374	0.022975

#### Computation time

One of the most important feature of the finite-difference scheme is the time spent for the problem solution. In Table 4 the computation time at using the CFDS and the scheme *Ros*_1_ is shown depending on the grid steps for the unchangeable problem parameters. Let us note that the computation time doesn’t exceed 1 second at the time step *τ* = 0.1 and any mesh steps *h* under consideration. However, these results possess poor accuracy. Increasing a grid node number leads to essentially increasing the computation time for the Rosenbrock method in comparison with computation time spent by the CFDS. The required accuracy of the numerical solution achieved at the grid steps *h* = *τ* = 0.01 for the CFDS while the Rosenbrock method provides the similar accuracy only at the grid steps equal *h* = *τ* = 0.001 (or less than this value), which leads to increasing the computation time by 2 order of magnitude.

**Table 4:** The computation time in dependence on the grid steps *τ*, *h* at using the finite-difference scheme based on the Rosenbrock method and the CFDS for the parameters *L*_*z*_ = 40.0, *t* = 10.0, *ψ* = 10.0, *γ* = 1.0.

*τ*	*h*	Rosenbrock method *Ros*_1_ (sec)	Conservative f-d scheme (sec)
0.05	0.1	0.28	0.10
	0.05	0.55	0.089
	0.01	2.85	1.53
	0.001	28.89	15.57
0.01	0.1	1.50	0.42
	0.05	2.84	2.57 (4 iterations)
	0.01	14.35	12.31 (4 iterations)
	0.001	145.39	123.05 (4 iterations)
0.001	0.1	13.75	4.15
	0.05	27.35	9.28 (2 iterations)
	0.01	132.50	25.93
	0.001	1454.13	338.52

It should be stressed, that the comparable computation time in Table 4 for both schemes at using the time step *τ* = 0.01 occurs because of the iteration process presence (${\tilde{\theta }}_{1}=0.001,{\tilde{\theta }}_{2}=0.00001$) for the CFDS and this requires certain iteration number, which is shown in the brackets of last column.

## The set of 1D Schrödinger equation

### Problem statement and invariants

The Rosenbrock method application for a solution of the Schrödinger equation set is considered for the classic problem of nonlinear optics—the second harmonic generation (SHG). Under the phase matching condition of the interacting waves, this problem is described by the set of the following dimensionless Schrödinger equations

$$\begin{array}{c} \begin{array}{c} \frac{\partial A_{1}}{\partial t}+ıD_{1}\frac{\partial^{2}A_{1}}{\partial z^{2}}+ı\eta A_{2}A_{1}^{*}+ı\gamma V\left(z\right)A_{1}=0,\\ \frac{\partial A_{2}}{\partial t}+ıD_{2}\frac{\partial^{2}A_{2}}{\partial z^{2}}+ı\eta A_{1}^{2}+ı\gamma V\left(z\right)A_{2}=0,\;0<z<L_{z},t>0 \end{array} \end{array}$$ (48)

with initial conditions

$$\begin{array}{c} A_{1}{{|}_{t=0}=A_{10}\left(z\right)=e^{-{\left(z-L_{z_{c}}\right)}^{2}+ı\chi \left(z-L_{z_{c}}\right)},\;A_{2}|}_{t=0}=0 \end{array}$$ (49)

and BCs

$$\begin{array}{c} A_{1}{{|}_{z=0,L_{z}}=A_{2}|}_{z=0,L_{z}}=0, \end{array}$$ (50)

for the finite distributions of the complex amplitudes. As we stressed above, often instead of BCs (50) one states that the complex amplitude and its derivatives in z-coordinate tends to zero at *z* → ∞:

$$\begin{array}{c} A_{j}{|}_{z\to \pm \infty }=0,j=1,2. \end{array}$$ (51)

In addition to the definitions introduced above, let us note, that in (48) *A*_1_ = *A*_1_(*t*, *z*), *A*_2_ = *A*_2_(*t*, *z*) are the complex amplitudes of the interacting laser pulses and the parameters *D*_1_, *D*_2_, *η* are the real coefficients.

The problem (48)–(50) has the well-known invariants (the conservation laws):

$$\begin{array}{c} I_{1}=\int_{0}^{L_{z}}\left(\right|A_{1}{|}^{2}+|A_{2}{|}^{2})dz=const \end{array}$$ (52)

is the first invariant(energy invariant);

$$\begin{array}{c} I_{3}=\int_{0}^{L_{z}}(\eta \left(A_{1}^{2}A_{2}^{*}+{\left(A_{1}^{*}\right)}^{2}A_{2}\right)-2D_{1}|\frac{\partial A_{1}}{\partial z}{|}^{2}-D_{2}|\frac{\partial A_{2}}{\partial z}{|}^{2}+\gamma V\left(z\right)\left(2\right|A_{1}{|}^{2}+|A_{2}{|}^{2})dz=const \end{array}$$ (53)

is the third invariant(Hamiltonian).

### The finite-difference scheme construction based on the Rosenbrock method for the Schrödinger equation set

As in previous consideration let’s represent the complex amplitudes by using real and imaginary parts

$$\begin{array}{c} A_{p}=u_{p}+ıv_{p},p=1,2, \end{array}$$ (54)

in nodes of the grid *ω*_*z*_ we define the following mesh functions

$$\begin{array}{c} \begin{array}{c} {\bar{A}}_{p,j}={\bar{A}}_{ph}\left(t,z_{j}\right)={\bar{u}}_{p,j}+ı{\bar{v}}_{p,j},\;{\bar{u}}_{p,j}={\bar{u}}_{ph}\left(t,z_{j}\right),\\ {\bar{v}}_{p,j}={\bar{v}}_{ph}\left(t,z_{j}\right),\;0\leq j\leq N_{z},p=1,2. \end{array} \end{array}$$ (55)

For the equation set (48) solution let’s write the following ODE set

$$\begin{array}{c} \frac{d\bar{U}\left(t\right)}{dt}=G\left(\bar{U}\right),j=\bar{1,N_{z}-1}, \end{array}$$ (56)

where the function $\bar{U}$ has the following form

$$\begin{array}{c} \bar{U}=({\bar{u}}_{1,0},{\bar{u}}_{1,1},...,{\bar{u}}_{1,N_{z}},{\bar{v}}_{1,0},{\bar{v}}_{1,1},...,{\bar{v}}_{1,N_{z}},{\bar{u}}_{2,0},{\bar{u}}_{2,1},...,{\bar{u}}_{2,N_{z}},{\bar{v}}_{2,0},{\bar{v}}_{2,1},...,{\bar{v}}_{2,N_{z}}), \end{array}$$

and the vector $G(\bar{U})$ computed as follows

$$\begin{array}{c} G(\bar{U})=(\begin{array}{c} D_{1}\Lambda_{\bar{z}z}{\bar{v}}_{1j}+\gamma V_{j}{\bar{v}}_{1j}+\eta \left({\bar{u}}_{1j}{\bar{v}}_{2j}-{\bar{u}}_{2j}{\bar{v}}_{1j}\right)\\ -D_{1}\Lambda_{\bar{z}z}{\bar{u}}_{1j}-\gamma V_{j}{\bar{u}}_{1j}-\eta \left({\bar{u}}_{1j}{\bar{u}}_{2j}+{\bar{v}}_{1j}{\bar{v}}_{2j}\right)\\ D_{2}\Lambda_{\bar{z}z}{\bar{v}}_{2j}+\gamma V_{j}{\bar{v}}_{2j}+2\eta {\bar{u}}_{1j}{\bar{v}}_{1j}\\ -D_{2}\Lambda_{\bar{z}z}{\bar{u}}_{2j}-\gamma V_{j}{\bar{u}}_{2j}-\eta \left({\bar{u}}_{1j}^{2}+{\bar{v}}_{1j}^{2}\right) \end{array}),j=\bar{1,N_{z}-1}. \end{array}$$ (57)

In (56) and (57) we do not consider boundary nodes because of using the zero-value BCs: ${\bar{u}}_{1,0}={\bar{u}}_{1,N_{z}}={\bar{v}}_{1,0}={\bar{v}}_{1,N_{z}}={\bar{u}}_{2,0}={\bar{u}}_{2,N_{z}}={\bar{v}}_{2,0}={\bar{v}}_{2,N_{z}}=0$.

Let us define the initial distribution of the complex amplitude at using the definitions introduced above

$$\begin{array}{c} {\bar{A}}_{1,j}{{|}_{t=0}=e^{-{\left(z_{j}-L_{z_{c}}\right)}^{2}+ı\chi \left(z_{j}-L_{z_{c}}\right)},\;{\bar{A}}_{2,j}|}_{t=0}=0. \end{array}$$ (58)

Now we introduce on the grid *ω*_*t*_ the following mesh functions

$$\begin{array}{c} \begin{array}{c} A_{k,m,j}=A_{ph}\left(t_{m},z_{j}\right)=u_{p,m,j}+ıv_{p,m,j},\;u_{k,m,j}=u_{ph}\left(t_{m},z_{j}\right),\\ v_{k,m,j}=v_{ph}\left(t_{m},z_{j}\right),\;0\leq j\leq N_{z},0\leq m\leq N_{t},p=1,2 \end{array} \end{array}$$ (59)

and vectors

$$\begin{array}{c} \begin{array}{cc} \hat{U}= & (u_{1,m+1,0},...,u_{1,m+1,N_{z}},v_{1,m+1,0},...,v_{1,m+1,N_{z}},\\ & u_{2,m+1,0},...,u_{2,m+1,N_{z}},v_{2,m+1,0},...,v_{2,m+1,N_{z}}),\\ U= & (u_{1,m,0},...,u_{1,m,N_{z}},v_{1,m,0},...,v_{1,m,N_{z}},u_{2,m,0},...,u_{2,m,N_{z}},v_{2,m,0},...,v_{2,m,N_{z}}). \end{array} \end{array}$$ (60)

Below, for brevity, we omit the index *h* in notation of the mesh functions.

The solution on the next time layer computed using (18), (19) with the function *G*(*U*) defined in (57). Function *G*_*U*_ is the corresponding Jacobian. The equation set is solved by using the Thomas algorithm, similar to case of the single Schrödinger Eqs (31) and (32). Obviously, at the solution of the Schrödinger equation set, the matrix dimension as well as vector dimension changes only.

### The CFDS construction for the Schrödinger equation set

Let us use the same grids, which are written above, and introduce the following definitions:

$$\begin{array}{c} \begin{array}{c} A_{p}=A_{p,j}=A_{p,m,j}=A_{p}\left(t_{m},z_{j}\right),\;\hat{A_{p}}={\hat{A}}_{p,j}={\hat{A}}_{p,m,j}=A_{p}\left(t_{m}+\tau ,z_{j}\right),\\ \overset{0.5}{A_{p}}=0.5\left({\hat{A}}_{p}+A_{p}\right),\;\overset{0.5}{A_{1}^{2}}=0.5\left({{\hat{A}}_{1}}^{2}+{A_{1}}^{2}\right),\;p=1,2. \end{array} \end{array}$$ (61)

Using these notations, we write for Eq (48) the following two-layers implicit scheme:

$$\begin{array}{c} \begin{array}{cc} \frac{{\hat{A}}_{1}-A_{1}}{\tau }+ıD_{1}\Lambda_{\bar{z}z}\overset{0.5}{A_{1}}+ı\eta \overset{0.5}{A_{1}^{*}}\overset{0.5}{A_{2}}+ı\gamma V\overset{0.5}{A_{1}}=0, & \\ \frac{{\hat{A}}_{2}-A_{2}}{\tau }+ıD_{2}\Lambda_{\bar{z}z}\overset{0.5}{A_{2}}+ı\eta \overset{0.5}{A_{1}^{2}}+ı\gamma V\overset{0.5}{A_{2}}=0, & j=\bar{1,N_{z}-1},m=\bar{0,N_{t}-1} \end{array} \end{array}$$ (62)

with initial conditions

$$\begin{array}{c} {A_{p,0}}_{j}=A_{p,0}\left(z_{j}\right),p=1,2 \end{array}$$ (63)

and BCs

$$\begin{array}{c} {\hat{A}}_{p,0}={\hat{A}}_{p,N_{z}}=0,p=1,2. \end{array}$$ (64)

Because the finite-difference scheme (62–64) is nonlinear one, we use for its solution the following iteration process:

$$\begin{array}{c} \begin{array}{cc} \frac{\overset{s+1}{{\hat{A}}_{1}}-A_{1}}{\tau }+ıD_{1}\Lambda_{\bar{z}z}\overset{s+1}{\overset{0.5}{A_{1}}}+ı\eta \overset{s}{{\overset{0.5}{A_{1}}}^{*}}\overset{s}{\overset{0.5}{A_{2}}}+ı\gamma V\overset{s+1}{\overset{0.5}{A_{1}}}=0, & \\ \frac{\overset{s+1}{{\hat{A}}_{2}}-A_{2}}{\tau }+ıD_{2}\Lambda_{\bar{z}z}\overset{s+1}{\overset{0.5}{A_{2}}}+ı\eta \overset{s}{{\overset{0.5}{A_{1}}}^{2}}+ı\gamma V\overset{s+1}{\overset{0.5}{A_{2}}}=0, & j=\bar{1,N_{z}-1},m=\bar{0,N_{t}-1}. \end{array} \end{array}$$ (65)

The BCs on iteration is written in the form

$$\begin{array}{c} \overset{s+1}{{\hat{A}}_{p,0}}=\overset{s+1}{{\hat{A}}_{p,N_{z}}}=0,p=1,2. \end{array}$$ (66)

The iteration process is stopped if the following inequality

$$\begin{array}{c} \underset{z_{j}}{max}|\overset{s+1}{\hat{A}}-\overset{s}{\hat{A}}|\leq {\tilde{\theta }}_{1}\underset{z_{j}}{max}\left|\overset{s}{\hat{A}}\right|+{\tilde{\theta }}_{2} \end{array}$$ (67)

is valid, where ${\tilde{\theta }}_{1},{\tilde{\theta }}_{2}>0$ are real constant.

The solution method of the difference Eq (65) is the similar to solution of the Eqs (41)–(44) considered above.

### Computer simulation results

The method efficiency we evaluate with using the norms:

$$\begin{array}{c} \begin{array}{ccc} \delta A_{12}= & \max_{q}\max_{1\leq j\leq N_{z}}\left|\right|{A_{q}}_{Ros_{1}}\left(t_{m},z_{j}\right){|}^{2}-\left|\right|{A_{q}}_{Ros_{2}}\left(t_{m},z_{j}\right){|}^{2}|, & \\ \delta A_{C1}= & \max_{q}\max_{1\leq j\leq N_{z}}\left|\right|{A_{q}}_{Ros_{1}}\left(t_{m},z_{j}\right){|}^{2}-|{A_{q}}_{C}\left(t_{m},z_{j}\right){|}^{2}|, & \\ \delta A_{C2}= & \max_{q}\max_{1\leq j\leq N_{z}}\left|\right|{A_{q}}_{Ros_{2}}\left(t_{m},z_{j}\right){|}^{2}-|{A_{q}}_{C}\left(t_{m},z_{j}\right){|}^{2}|, & q=1,2, \end{array} \end{array}$$ (68)

which characterize the corresponding solution deviations from each other.

#### The illustration of Rosenbrock scheme conditional conservatism for the set of 1D Schrödinger equations

The conditional conservatism of the finite-difference scheme based on the Rosenbrock method, is demonstrated for the parameters $L_{z}=40.0,L_{z_{c}}=20.0,D_{1}=D_{2}=1.0,\chi =0$ and the following parameters of the potential *L*_*v*_ = 20.0, *m*_*v*_ = 2 at time moment *t* = 1.0 and for the mesh steps *h*_*z*_ = *τ* = 0.01. At initial time moment the complex amplitude distribution is defined by (50). The obtained results show that changing of the first invariant *I*_1_ for the CFDS is at least an order of magnitude less in comparison with the corresponding value for the Rosenbrock method. But in contrast to the Rosenbrock method, it does not depend on the parameters *γ* and *η* and causes by the iteration process presence. It should be stressed, that the Rosenbrock method accuracy at its using with the complex parameter *β* = 0.5 + 0.5*ı* is worse for *γ* = −1 and for the parameter *η*, which is less or equal to 10. With further increasing the parameter *η*, the Hamiltonian deviation from the initial value increases significantly and this violation reaches about 10%−24% depending on the parameters *η*, *γ* values and on the parameter *β* (it is a complex one or a real one) value in the Rosenbrock method. To achieve the invariant conservation with acceptable accuracy and the similar solution accuracy as takes place for the CFDS it is necessary to decrease the grid steps at least tenfold at using the finite-difference scheme based on the Rosenbrock method. Moreover, with increasing the time interval, during which the computation is provided, the mesh steps have to decrease also.

**Fig 8:**
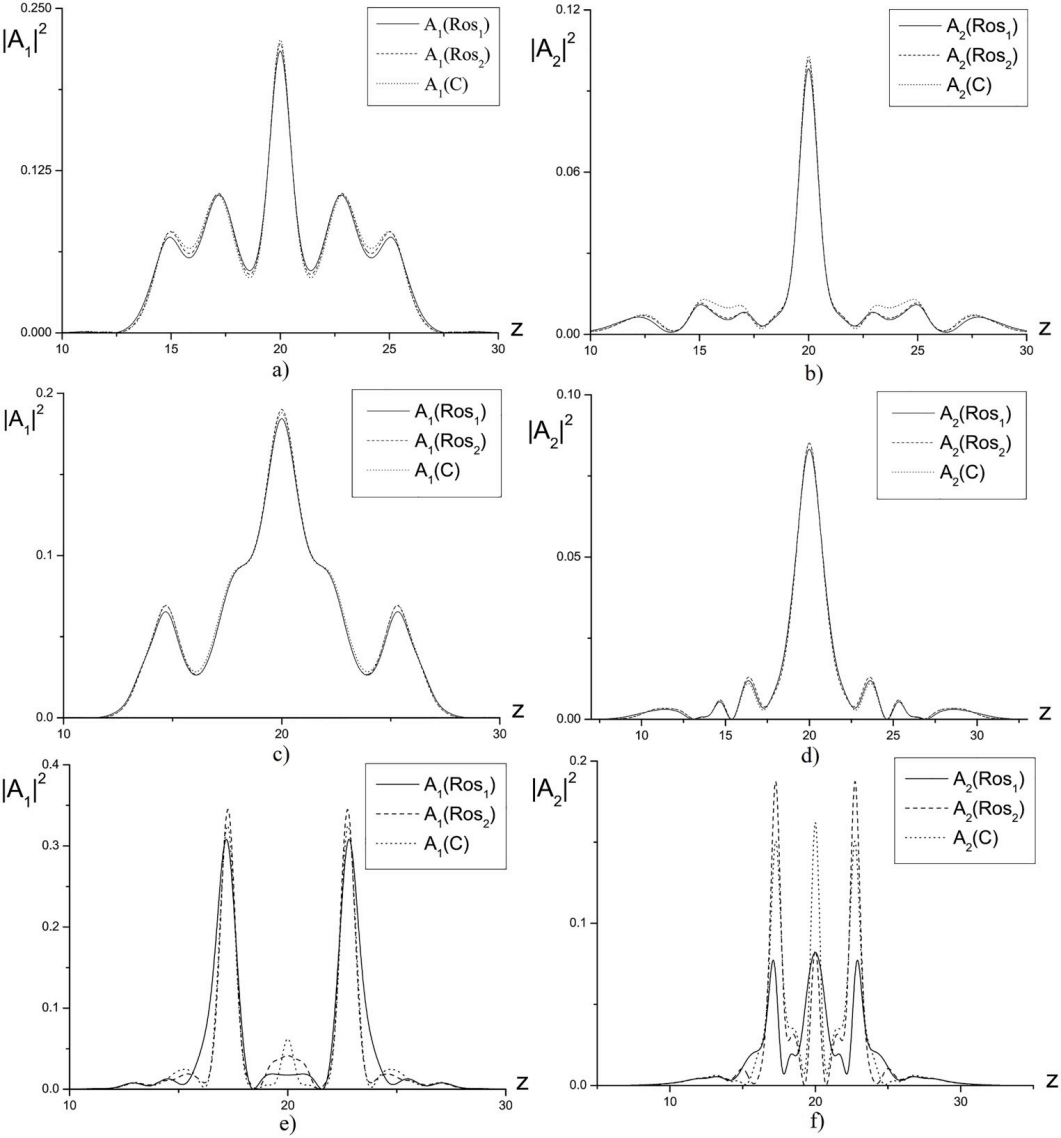
Intensity profile for waves with basic (a,c,e) and the doubled (b,d,f) frequency at the time moment *t* = 1.0 and computed using various finite-difference schemes for the parameters *γ* = 0(*a*, *b*);1(*c*, *d*);−1(*e*, *f*) and *η* = 20.

As an example, in Fig 8 the intensity profiles, computed using three finite-difference schemes under consideration, are depicted. This Figure illustrates also the essential influence of the parameter *γ* on the solution accuracy. Let us note, that the maximal difference in the wave intensity profiles achieves for *γ* = −1. Exactly, the significant deviation of the Hamiltonian for the Rosenbrock method is observed. It should be stressed that with decreasing the time step until *τ* = 10^−3^ the solution obtained using the Rosenbrock method coincides with the solution obtained using the CFDS. Essentially, that the solutions, obtained using the CFDS for the time steps *τ* = 10^−2^ and *τ* = 10^−3^, differ each other within the approximation order.

#### Computation time in dependence of grid steps

The computation time dependence on the grid steps for the two wave interaction with the parameters $L_{z_{c}}=20.0,D_{1}=D_{2}=1.0,\eta =20,\gamma =1,\chi =0$ and the following parameters of the potential *L*_*v*_ = 20.0, *m*_*v*_ = 2 for the time and spatial intervals *L*_*t*_ = 10 and *L*_*z*_ = 40 correspondingly is shown in Table 5. Computer simulation is provided for the initial Gaussian distribution of complex amplitude for the first wave. The second wave has zero-value of its amplitude in initial time moment. Let us note, that at *h* = 0.1 and any time steps *τ* under consideration, the computation time for the both finite-difference schemes doesn’t exceed 1 second. With increasing the grid nodes number, the computation time increases significantly at using the finite-difference scheme based on Rosenbrock method in contrast to the corresponding value for using the CFDS. It should be kept in mind, that the sufficient numerical solution accuracy for the CFDS achieves already for the grid steps *h* = *τ* = 0.01. At the same time, the similar accuracy at using the Rosenbrock method takes place if the grid steps are decreased at least tenfold.

**Table 5:** The computation time dependence on the grid steps *τ*, *h* at using the finite-difference scheme based on the Rosenbrock method (*Ros*_1_) and the CFDS.

*τ*	*h*	Rosenbrock method *Ros*_1_ (sec)	Conservative f-d scheme (sec)
0.1	0.05	1	1
	0.01	1	1
	0.001	8	10
0.05	0.05	1	1
	0.01	2	1
	0.001	16	5
0.01	0.05	2	1
	0.01	9	3
	0.001	87	20
0.001	0.05	18	3
	0.01	89	12
	0.001	902	133

## The finite-difference scheme based on the Rosenbrock method with ABCs

### Problem statement for 1D nonlinear Schrödinger equation

Let us consider the 1D nonlinear Schrödinger equation

$$\begin{array}{c} \frac{\partial A}{\partial t}+ıD_{z}\frac{\partial^{2}A}{\partial z^{2}}+ı\phi A+ı\psi {\left|A\right|}^{2}A=0,t>0,0<z<L_{z}, \end{array}$$ (69)

which describe the light pulse propagation in 1D Photonic crystal (PC). In this case, the parameters *D*_*z*_, *ϕ* defined as: $D_{z}=\frac{1}{4\pi \chi },\phi =\pi \chi$ and relate to the initial complex amplitude distribution [3]

$$\begin{array}{c} {A|}_{t=0}=e^{-{\left(z-L_{z_{c}}\right)}^{2}+ı2\pi \chi \left(z-L_{z_{c}}\right)}, \end{array}$$ (70)

corresponding to the wave propagation along the z-axis and BCs for the Eq (69) are written in the following way

$$\begin{array}{c} \begin{array}{c} {(\frac{\partial A}{\partial t}-2D_{z}\Omega_{z}\frac{\partial A}{\partial z}+2ı\phi A+ı\psi {\left|A\right|}^{2}A)|}_{z=L_{0}}=0,\\ {(\frac{\partial A}{\partial t}+2D_{z}\Omega_{z}\frac{\partial A}{\partial z}+2ı\phi A+ı\psi {\left|A\right|}^{2}A)|}_{z=L_{z}}=0, \end{array} \end{array}$$ (71)

It should be stressed that in (71) the parameter *Ω*_*z*_ is defined by the parameter *χ* of the initial distribution (70) as *Ω*_*z*_ = 2*πχ*. We see that this equation differs from the problem (1) by term related to the potential *V* and a presence of the relation between parameters *D*_*z*_ and *ϕ*.

Note, that the BCs (71) were proposed in [3] for computer simulation of the femtosecond pulse propagation in a nonlinear PC during the time interval about several thousand dimensionless units, which is very long time interval in comparison with the initial pulse duration being equal to a few dimensionless units. The PC occupies a small part of z-axis spatial domain.

### Finite-difference scheme construction for Rosenbrock method

In contrast to the zero-value BCs, the equations for nodes *j* = 0, *N*_*z*_ of the grid *ω*_*z*_ (see (9)) in the case under consideration are written in following form

$$\begin{array}{c} \frac{d\bar{U}\left(t\right)}{dt}=G\left(\bar{U}\right),\;j=\left\{0,N_{z}\right\}, \end{array}$$ (72)

where $\bar{U}=({\bar{u}}_{0},{\bar{v}}_{0},{\bar{u}}_{N_{z}},{\bar{v}}_{N_{z}})$, and the vector $G(\bar{U})$ is computed as follows

$$\begin{array}{c} G(\bar{U})=(\begin{array}{c} 2D_{z}\Omega_{z}\frac{u_{1}-u_{0}}{h}+2\phi v_{0}+\psi \left({u_{0}}^{2}+{v_{0}}^{2}\right)v_{0}\\ 2D_{z}\Omega_{z}\frac{v_{1}-v_{0}}{h}-2\phi u_{0}-\psi \left({u_{0}}^{2}+{v_{0}}^{2}\right)u_{0}\\ -2D_{z}\Omega_{z}\frac{u_{N_{z}}-u_{N_{z}-1}}{h}+2\phi v_{N_{z}}+\psi \left({u_{N_{z}}}^{2}+{v_{N_{z}}}^{2}\right)v_{N_{z}}\\ -2D_{z}\Omega_{z}\frac{v_{N_{z}}-v_{N_{z}-1}}{h}-2\phi u_{N_{z}}-\psi \left({u_{N_{z}}}^{2}+{v_{N_{z}}}^{2}\right)u_{N_{z}} \end{array}). \end{array}$$ (73)

After introducing the mesh along time coordinate we need to add the difference equation for the node *j* = *N*_*z*_ in the equation set (29):

$$\begin{array}{c} -Q_{N_{z}}{\hat{Y}}_{N_{z}-1}+C_{N_{z}}{\hat{Y}}_{N_{z}}=F_{N_{z}}\left(U_{N_{z}}\right),\;{\hat{Y}}_{N_{z}}={\left(k_{u_{N_{z}}},k_{v_{N_{z}}},{\tilde{k}}_{u_{N_{z}}},{\tilde{k}}_{v_{N_{z}}}\right)}^{T}, \end{array}$$ (74)

instead of zero-value of the vector $\hat{Y}$ defined in (31) for this mesh node. Here, $Q_{N_{z}},C_{N_{z}}$ are matrices with the following components

$$\begin{array}{c} C_{N_{z}}=(\begin{array}{cccc} c_{1} & c_{2} & c_{3} & c_{4}\\ c_{5} & c_{6} & c_{7} & c_{8}\\ c_{9} & c_{10} & c_{11} & c_{12}\\ c_{13} & c_{14} & c_{15} & c_{16} \end{array}), \end{array}$$

$$\begin{array}{c} c_{1}=c_{11}=1+2D_{z}\Omega_{z}\frac{\beta_{R}\tau }{h}-2\tau \beta_{R}\psi u_{N_{z}}v_{N_{z}};c_{5}=c_{15}=\beta_{R}\tau \left(2\phi +\psi v_{N_{z}}^{2}+3\psi u_{N_{z}}^{2}\right);\\ c_{2}=c_{12}=-\beta_{R}\tau \left(2\phi +\psi u_{N_{z}}^{2}+3\psi v_{N_{z}}^{2}\right);c_{6}=c_{16}=1+2D_{z}\Omega_{z}\frac{\beta_{R}\tau }{h}+2\beta_{R}\tau \psi u_{N_{z}}v_{N_{z}};\\ c_{3}=-c_{9}=-2D_{z}\Omega_{z}\frac{\beta_{I}\tau }{h}+2\tau \psi u_{N_{z}}v_{N_{z}};c_{7}=-c\left(13\right)=-\tau \beta_{I}\left(2\phi +\psi v_{N_{z}}^{2}-3\psi u_{N_{z}}^{2}\right);\\ c_{4}=-c_{10}=\beta_{I}\tau \left(2\phi +\psi u_{N_{z}}^{2}+3\psi v_{N_{z}}^{2}\right);c_{8}=-c\left(14\right)=-2D_{z}\Omega_{z}\frac{\beta_{I}\tau }{h}-2\tau \beta_{I}\psi u_{N_{z}}v_{N_{z}}; \end{array}$$

$$\begin{array}{c} Q_{N_{z}}=(\begin{array}{cccc} 2D_{z}\Omega_{z}\frac{\beta_{R}\tau }{h} & 0 & -2D_{z}\Omega_{z}\frac{\beta_{I}\tau }{h} & 0\\ 0 & 2D_{z}\Omega_{z}\frac{\beta_{R}\tau }{h} & 0 & -2D_{z}\Omega_{z}\frac{\beta_{I}\tau }{h}\\ 2D_{z}\Omega_{z}\frac{\beta_{I}\tau }{h} & 0 & 2D_{z}\Omega_{z}\frac{\beta_{R}\tau }{h} & 0\\ 0 & 2D_{z}\Omega_{z}\frac{\beta_{I}\tau }{h} & 0 & 2D_{z}\Omega_{z}\frac{\beta_{R}\tau }{h} \end{array}), \end{array}$$

$$\begin{array}{c} F_{N_{z}}=(\begin{array}{c} -2D_{z}\Omega_{z}\frac{u_{N_{z}}-u_{N_{z}-1}}{h}+2\phi v_{N_{z}}+\psi \left({u_{N_{z}}}^{2}+{v_{N_{z}}}^{2}\right)v_{N_{z}}\\ -2D_{z}\Omega_{z}\frac{v_{N_{z}}-v_{N_{z}-1}}{h}-2\phi u_{N_{z}}-\psi \left({u_{N_{z}}}^{2}+{v_{N_{z}}}^{2}\right)u_{N_{z}}\\ 0\\ 0 \end{array}). \end{array}$$

As a result, the vector $\hat{Y}$ in the grid node *j* = *N*_*z*_ is written in the following way

$$\begin{array}{c} {\hat{Y}}_{N_{z}}=\beta_{N_{z}+1}={\left(C_{N_{z}}-Q_{N_{z}}\alpha_{N_{z}}\right)}^{-1}(F_{N_{z}}+Q_{N_{z}}\beta_{N_{z}}). \end{array}$$ (75)

The BC in the grid node *j* = 0 is written similarly to (74):

$$\begin{array}{c} -B_{0}{\hat{Y}}_{1}+C_{0}{\hat{Y}}_{0}=F_{0}\left(U_{0}\right),\;{\hat{Y}}_{0}={\left(k_{u_{0}},k_{v_{0}},{\tilde{k}}_{u_{0}},{\tilde{k}}_{v_{0}}\right)}^{T}, \end{array}$$ (76)

which is also added to the equation set (29) for the mesh node *j* = 0. Obviously, in (76) the matrices *B*_0_, *C*_0_ have the following components

$$\begin{array}{c} C_{0}=(\begin{array}{cccc} c_{1} & c_{2} & c_{3} & c_{4}\\ c_{5} & c_{6} & c_{7} & c_{8}\\ c_{9} & c_{10} & c_{11} & c_{12}\\ c_{13} & c_{14} & c_{15} & c_{16} \end{array}), \end{array}$$

$$\begin{array}{c} c_{1}=c_{11}=1+2D_{z}\Omega_{z}\frac{\beta_{R}\tau }{h}-2\tau \beta_{R}\psi u_{0}v_{N_{z}};\;c_{2}=c_{12}=-\beta_{R}\tau \left(2\phi +\psi u_{0}^{2}+3\psi v_{0}^{2}\right);\\ c_{3}=-c_{9}=-2D_{z}\Omega_{z}\frac{\beta_{I}\tau }{h}+2\tau \psi u_{0}v_{0};\;c_{4}=-c_{10}=\beta_{I}\tau \left(2\phi +\psi u_{0}^{2}+3\psi v_{0}^{2}\right);\\ c_{5}=c_{15}=\beta_{R}\tau \left(2\phi +\psi v_{0}^{2}+3\psi u_{0}^{2}\right);\;c_{6}=c_{16}=1+2D_{z}\Omega_{z}\frac{\beta_{R}\tau }{h}+2\beta_{R}\tau \psi u_{0}v_{0};\\ c_{7}=-c\left(13\right)=-\tau \beta_{I}\left(2\phi +\psi v_{0}^{2}-3\psi u_{0}^{2}\right);\;c_{8}=-c\left(14\right)=-2D_{z}\Omega_{z}\frac{\beta_{I}\tau }{h}-2\tau \beta_{I}\psi u_{0}v_{0}; \end{array}$$

$$\begin{array}{c} B_{0}=(\begin{array}{cccc} 2D_{z}\Omega_{z}\frac{\beta_{R}\tau }{h} & 0 & -2D_{z}\Omega_{z}\frac{\beta_{I}\tau }{h} & 0\\ 0 & 2D_{z}\Omega_{z}\frac{\beta_{R}\tau }{h} & 0 & -2D_{z}\Omega_{z}\frac{\beta_{I}\tau }{h}\\ 2D_{z}\Omega_{z}\frac{\beta_{I}\tau }{h} & 0 & 2D_{z}\Omega_{z}\frac{\beta_{R}\tau }{h} & 0\\ 0 & 2D_{z}\Omega_{z}\frac{\beta_{I}\tau }{h} & 0 & 2D_{z}\Omega_{z}\frac{\beta_{R}\tau }{h} \end{array}), \end{array}$$

$$\begin{array}{c} F_{0}=(\begin{array}{c} 2D_{z}\Omega_{z}\frac{u_{1}-u_{0}}{h}+2\phi v_{0}+\psi \left({u_{0}}^{2}+{v_{0}}^{2}\right)v_{0}\\ 2D_{z}\Omega_{z}\frac{v_{1}-v_{0}}{h}-2\phi u_{0}-\psi \left({u_{0}}^{2}+{v_{0}}^{2}\right)u_{0}\\ 0\\ 0 \end{array}). \end{array}$$

Consequently, we write the mesh function in the zero node as

$$\begin{array}{c} {\hat{Y}}_{0}=\alpha_{1}{\hat{Y}}_{1}+\beta_{1},\;\alpha_{1}={C_{0}}^{-1}B_{0},\;\beta_{1}={C_{0}}^{-1}F_{0}\left(U_{0}\right), \end{array}$$ (77)

instead of zero-value of the vector $\hat{Y}$ defined in (31) for this mesh node. The matrix *α*_1_ and the vector *β*_1_ defined in (77) are used for the Thomas algorithm (32).

### Approximation of the ABCs for the CFDS

To do a comparison of both finite-difference schemes we write below an approximation of the ABCs (71) for the CFDS (34)

$$\begin{array}{c} \begin{array}{c} \frac{{\hat{U}}_{0}-U_{0}}{\tau }-2D_{z}\Omega_{z}\frac{{\overset{0.5}{U}}_{1}-{\overset{0.5}{U}}_{0}}{h}+2ı\phi {\overset{0.5}{U}}_{0}+ı\psi {\left|{\overset{0.5}{U}}_{0}\right|}^{2}{\overset{0.5}{U}}_{0}=0,\\ \frac{{\hat{U}}_{N_{z}}-U_{N_{z}}}{\tau }+2D_{z}\Omega_{z}\frac{{\overset{0.5}{U}}_{N_{z}}-{\overset{0.5}{U}}_{N_{z}-1}}{h}+2ı\phi {\overset{0.5}{U}}_{N_{z}}+ı\psi {\left|{\overset{0.5}{U}}_{N_{z}}\right|}^{2}{\overset{0.5}{U}}_{N_{z}}=0,m=\bar{0,N_{t}-1}. \end{array} \end{array}$$ (78)

Since the difference Eq (78) are nonlinear ones, for its solution we use the iteration process

$$\begin{array}{c} \begin{array}{c} \frac{{\overset{s+1}{\hat{U}}}_{0}-U_{0}}{\tau }-2D_{z}\Omega_{z}\frac{{\overset{s+1}{\overset{0.5}{U}}}_{1}-{\overset{s+1}{\overset{0.5}{U}}}_{0}}{h}+2ı\phi {\overset{s+1}{\overset{0.5}{U}}}_{0}+ı\psi |{\overset{s}{\overset{0.5}{U}}}_{0}{|}^{2}{\overset{s}{\overset{0.5}{U}}}_{0}=0,\\ \frac{{\overset{s+1}{\hat{U}}}_{N_{z}}-U_{N_{z}}}{\tau }+2D_{z}\Omega_{z}\frac{{\overset{s+1}{\overset{0.5}{U}}}_{N_{z}}-{\overset{s+1}{\overset{0.5}{U}}}_{N_{z}-1}}{h}+2ı\phi {\overset{s+1}{\overset{0.5}{U}}}_{N_{z}}+ı\psi {\overset{s}{|\overset{0.5}{U}}}_{N_{z}}{|}^{2}{\overset{s}{\overset{0.5}{U}}}_{N_{z}}=0,m=\bar{0,N_{t}-1}. \end{array} \end{array}$$ (79)

Other formulae are written in the similar way to the difference Eq (34): see (37), (38).

### Computer simulation results

**Fig 9:**
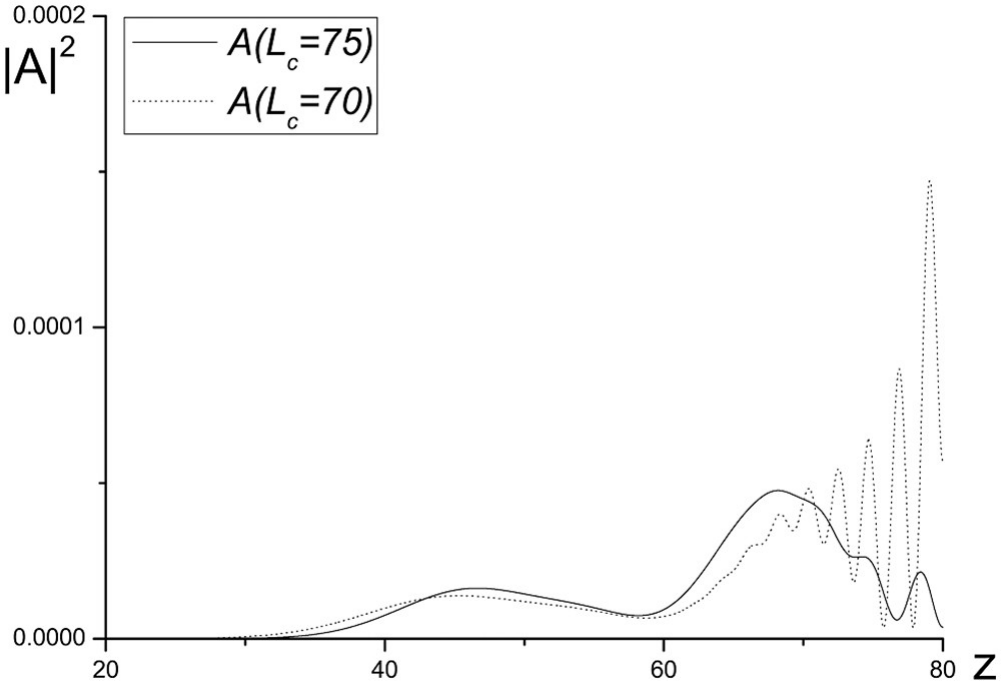
The intensity profile |*A*|^2^, computed for the parameters *ϕ* = 2.5, *χ* = 0.8, *D*_*z*_ = 0.1, *ψ* = 0 and mesh steps *h* = *τ* = 0.01 at the time moment, when the wave reflected from the artificial boundary, stated in section *L*_*z*_ = 80, achieves the section *z* = 30. **Two initial positions of the pulse are equal to** $L_{z_{c}}=70.0$ **(solid line)**, 75.0 **(dotted line)**.

Let us consider the problem with zero-value BC at the left boundary of the domain on z-coordinate and ABC at the right boundary. It is stated in the medium section *L*_*z*_ = 20 (except the Fig 9, where ABCs is stated in the section *L*_*z*_ = 80). The computer simulation is performed for the parameters *D*_*z*_ = 0.0796, *ϕ* = 3.14, *χ* = 1.0.

The computer simulation results are compared, in particular, with the analytical solution of the linear problem (69) (*ψ* = 0). Let us define the analytical solution as *A*_*Ex*_

$$\begin{array}{c} A_{Ex}=\frac{1}{f^{0.5}}e^{-\frac{{\left(z-L_{z_{c}}-\chi t\right)}^{2}}{f^{2}}-ı\frac{{\left(z-L_{z_{c}}-\chi t\right)}^{2}}{4D_{z}f}\frac{df}{dt}},f=\sqrt{1+{\left(4D_{z}t\right)}^{2}}. \end{array}$$ (80)

In Table 6 the maximal intensity differences between the numerical solution of the Eq (69), obtained using the finite-difference scheme based on the Rosenbrock method with various parameter *β*, and the numerical solution, obtained using the CFDS, and the analytical solution (80) are demonstrated. As it follows from the Table 6, the numerical solutions of the linear problem with the BCs under consideration differ insignificantly from each other. For example, the solutions coincide with the accuracy of magnitude order 10^−5^ at the steps *h* = *τ* = 0.01.

**Table 6:** The maximal intensity difference between the numerical solution, obtained using the finite-difference schemes based on the Rosenbrock methods (correspondingly, one denotes as *δA*_1*Ex*_ and *δA*_2*Ex*_) or based on the CFDS (*δA*_*CEx*_), and the analytical solution at various time moments.

*τ*	*h*	*t*	*δA*_1*Ex*_	*δA*_2*Ex*_	*δA*_*CEx*_
0.1	0.01	5	0.013016	0.013615	0.013652
		7.5	0.007288	0.005552	0.005557
		10	0.001212	0.000885	0.000886
0.01	0.1	5	0.175465	0.175663	0.175658
		7.5	0.02431	0.024387	0.02386
		10	0.009347	0.009351	0.009351
	0.01	5	0.012448	0.012436	0.012497
		7.5	0.003905	0.003954	0.003961
		10	0.000598	0.000606	0.000612
	0.001	5	0.005065	0.005094	0.005937
		7.5	0.003035	0.003024	0.003211
		10	0.000555	0.000563	0.000557
0.001	0.01	5	0.01244	0.01244	0.0124572
		7.5	0.003938	0.0039378	0.00393942
		10	0.0006034	0.00060347	0.0006035

In Fig 9 the dependence of the reflected wave intensity, appeared at the boundary with ABC, on the initial complex amplitude distribution center position is shown at computation using the finite-difference scheme based on the Rosenbrock method with the complex parameter $\beta =\frac{1}{2}\left(1+ı\right)$. The center of complex amplitude initial distribution is located in the section $L_{z_{c}}=70,75$, correspondingly. Thus, with decreasing the distance between initial distribution center and the section of the boundary with ABC, the reflected wave intensity decreases. This is showed in Fig 9. With increasing the distance between the pulse center and the section, in which the ABCs are stated, is a beam distraction influence increases. We see clearly that the reflected wave intensities do not exceed the theoretical assessments.

**Fig 10:**
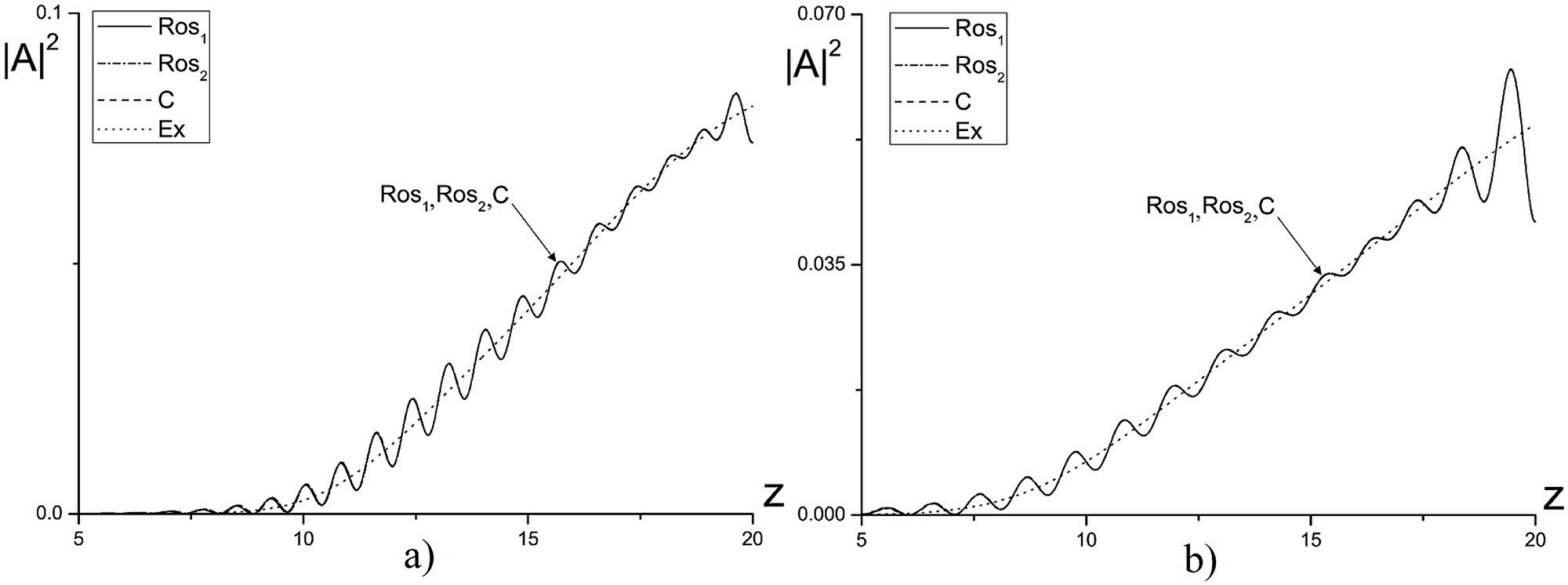
The intensity profile |*A*|^2^, computed using the finite-difference scheme based on the Rosenbrock methods (*Ros*_1_, *Ros*_2_) and the CFDS (*C*) as well as the standard solution (*Ex*), is depicted at the time moments *t* = 10.0(*a*), 20.0(*b*) and for the parameters *ϕ* = 2.5, *χ* = 0.8, *D*_*z*_ = 0.1, *ψ* = −5.0 and the mesh steps *h* = *τ* = 0.01.

In Fig 10 the intensity profile of the laser pulse, propagating in a medium with negative nonlinear lens *ψ* = −5.0 (defocusing medium), is depicted for a time moment when the laser pulse mainly transmitted through the artificial boundary. The essential result is that all numerical solutions, obtained using three finite-difference schemes under consideration, coincide (the maximal difference between solutions is about 3 ⋅ 10^−4^) and the reflected wave intensity is equal to about 0.01 in the time moment *t* = 10 and it is less than 0.005 in the time moment *t* = 20. We believe that the intensity oscillation is caused by constancy of the parameter *Ω*_*z*_ while this parameter can significantly deviate from the local wave number of the optical radiation near the artificial boundary (this is confirmed in Fig 9). It should be stressed, that we use the numerical solution, obtained on the basis of the CFDS with zero-value BCs in enough big domain, as the standard solution. We denote this solution with index *Ex*.

**Fig 11:**
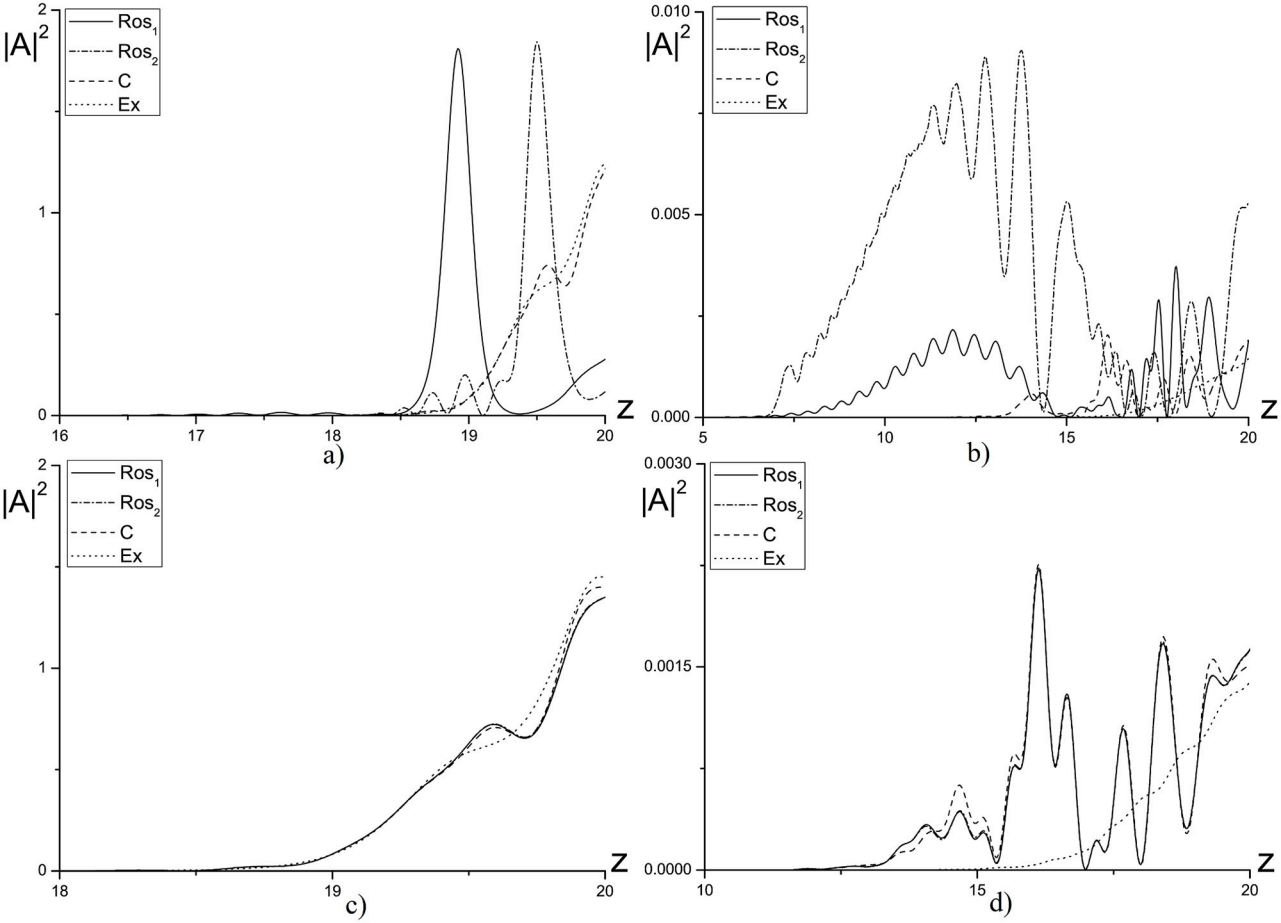
The intensity profile |*A*|^2^ at the time moment *t* = 5.0(*a*, *c*); 7.5(*b*, *d*), computed for the parameters *ϕ* = 2.5, *χ* = 0.8, *D*_*z*_ = 0.1, *ψ* = 5.0 and the mesh steps *τ* = 0.01(*a*, *b*); 0.001(*c*, *d*).

In Fig 11 the intensity profile for the pulse propagating in the self-focusing medium (*ψ* = 5.0) is shown. In Fig 11a and 11b the numerical solution deviation, obtained using the Rosenbrock methods for the time step *τ* = 0.01, from the exact solution becomes equal to about unity. With decreasing the time step to *τ* = 0.001 (see Fig 11c and 11d), the intensity profiles, obtained using considered finite-difference schemes, become much closer each other. It is important to stress that after main part of the light energy transmitted through the artificial boundary, the reflected intensity becomes less than 0.002, which is comparable with the approximation order of the finite-difference scheme.

Thus, we can conclude if we analyze the optical radiation propagation in defocusing medium, then the numerical solution, obtained using the finite-difference scheme based on the Rosenbrock method, approximates an exact solution with the same accuracy order as the CFDS. In the case of the self-focusing medium it is necessary to decrease the mesh step in time coordinate more than ten times at computation using the finite-difference scheme based on the Rosenbrock method to achieve the solution accuracy comparable with the accuracy of the solution obtained using the CFDS.

A similar investigation was made for the ABCs located at left boundary of the domain.

## Combined method

As it is well-known, the CFDS implementation for a nonlinear problem requires to use the iteration process. The Rosenbrock method is explicit one, but it has the conditional conservatism property. Therefore, we propose the combined method based on both methods to realize their advantages. At some propagation distance of the laser pulse, the combined method is more preferable in comparison with other methods. Another very important feature of the combined method consists in a type of the BCs for the problem (34): instead the ABCs (78) we state the Dirichlet BC. The ABCs (72)–(74) are stated only for the finite-difference scheme based on the Rosenbrock method.

The method is illustrated by the problem of the femtosecond laser pulse propagation in the PC (the problem (69)–(71)).

### Finite-difference scheme construction for the combined method

**Fig 12:**
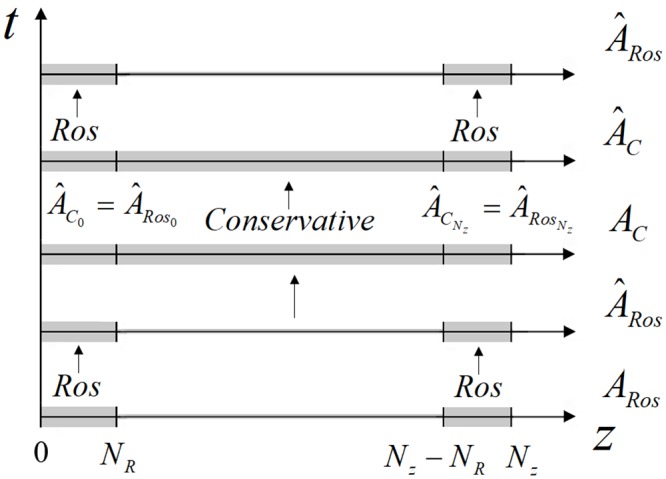
The combined method scheme.

The main idea of the combined method consists in using the Rosenbrock’s method near the boundaries of the domain. It means, that we introduce two sub-domains near left and right boundaries of the domain under consideration. We denote a number of the grid nodes as *N*_*R*_ (see Fig 12). In other part of the domain in z-coordinate, the CFDS is used for a computation. The problem solution is provided in several stages. In the first stage, the problem solution is computed in the sub-domains [0, *N*_*R*_] and [*N*_*z*_ − *N*_*R*_, *N*_*z*_] at using the finite-difference scheme based on the Rosenbrock method with ABCs. To write the finite-difference scheme let’s denote the solution, obtained in this stage, as ${\hat{A}}_{Ros}$ and the solution at the previous time step is denoted as *A*_*Ros*_. Then, the finite-difference scheme, corresponding to the first stage, is written in the following form:

$$\begin{array}{c} {\hat{A}}_{Ros}=A_{Ros}+\tau_{R}Re\bar{k},\;j=\bar{0,N_{R}};\bar{N_{z}-N_{R},N_{z}},m=\bar{0,N_{t}-1} \end{array}$$ (81)

where $Re\bar{k}$, as it was introduced above, is a real part of solution for the linear equation set, $\tau_{R}=\frac{\tau }{M}$ (*M* is an integer number) is a time step used for computation in sub-domains

$$\begin{array}{c} \begin{array}{cc} \left(E-\beta \tau_{R}G_{U}\right)\bar{k}=G\left(U\right), & \;j=\bar{1,N_{R}};\bar{N_{z}-N_{R},N_{z}-1},\\ \left(E-\beta \tau_{R}G_{U_{bound}}\right)\bar{k}=G\left(U_{bound}\right), & \;j=0,N_{z},m=\bar{0,N_{t}-1}. \end{array} \end{array}$$ (82)

Above we use the introduced definitions, $G_{U_{bound}}$ is the Jacobian of the right part vector *G*(*U*_*bound*_), which corresponds to the ABCs (73) and is written in the following form

$$\begin{array}{c} G(U_{bound})=(\begin{array}{c} 2D_{z}\Omega_{z}\frac{u_{1}-u_{0}}{h}+2\phi v_{0}+\psi \left({u_{0}}^{2}+{v_{0}}^{2}\right)v_{0}\\ 2D_{z}\Omega_{z}\frac{v_{1}-v_{0}}{h}-2\phi u_{0}-\psi \left({u_{0}}^{2}+{v_{0}}^{2}\right)u_{0}\\ -2D_{z}\Omega_{z}\frac{u_{N_{z}}-u_{N_{z}-1}}{h}+2\phi v_{N_{z}}+\psi \left({u_{N_{z}}}^{2}+{v_{N_{z}}}^{2}\right)v_{N_{z}}\\ -2D_{z}\Omega_{z}\frac{v_{N_{z}}-v_{N_{z}-1}}{h}-2\phi u_{N_{z}}-\psi \left({u_{N_{z}}}^{2}+{v_{N_{z}}}^{2}\right)u_{N_{z}} \end{array}). \end{array}$$ (83)

In formula (83) the functions *u* and *v* denote the real and imaginary parts of the solution *A*_*Ros*_. As it mentioned above, the set of equations (81)–(83) is solved by using the Thomas algorithm.

The second stage consists in the problem solution by using the CFDS with BCs defined by the mesh function ${\hat{A}}_{Ros}$. Let us denote the problem solution on this stage as ${\hat{A}}_{C}$ and the solution on the previous time step is denoted as *A*_*C*_. Thus, the difference problem on the second stage is written as follow:

$$\begin{array}{c} \frac{\overset{s+1}{{\hat{A}}_{C}}-A_{C}}{\tau }+ıD_{z}\Lambda_{\bar{z}z}\overset{s+1}{\overset{0.5}{A_{C}}}+ı\phi \overset{s+1}{\overset{0.5}{A_{C}}}+ı\psi {\left|\overset{s}{\overset{0.5}{A_{C}}}\right|}^{2}\overset{s}{\overset{0.5}{A_{C}}}=0,j=\bar{1,N_{z}-1},m=\bar{0,N_{t}-1}, \end{array}$$ (84)

with BCs:

$$\begin{array}{c} \overset{s+1}{{\hat{A}}_{C_{0}}}={\hat{A}}_{Ros_{0}},\;\overset{s+1}{{\hat{A}}_{C_{N_{z}}}}={\hat{A}}_{Ros_{N_{z}}} \end{array}$$ (85)

with corresponding condition of the iteration process stopping (40) and with choosing the difference functions on the zero-value iteration.

After this, we use the mesh function ${\hat{A}}_{C}$ as the initial condition for a computation of the problem solution on the next time layer in the sub-domains [0, *N*_*R*_] and [*N*_*z*_ − *N*_*R*_, *N*_*z*_] at using the finite-difference scheme based on the Rosenbrock method. With this aim we define the mesh function *A*_*Ros*_ for the Rosenbrock method as

$$\begin{array}{c} A_{Ros}={\hat{A}}_{C},j=\bar{0,N_{R}};\bar{N_{z}-N_{R},N_{z}},m=\bar{0,N_{t}-1} \end{array}$$ (86)

and using the finite-difference scheme written in (81)–(83), the computation is performed. Then the problem (84) and (85) is solved at new time layer and the process repeats. Note, that for finding the solution at the first time layer we use the initial complex amplitude distribution.

Thus, we obtain the implicit combined method for the nonlinear Schrödinger equation solution (Fig 12).

### Computer simulation results

Let us consider the computer simulation results obtained using the finite-difference scheme based on the Rosenbrock method with the parameter *β* = 0.5(1 + *ı*) (denote with index *Ros*) or on the CFDS (denote with index *C*) or on the combined method (denotes with index *R*&*C*) (with parameter *β* = 0.5). We compare the obtained results between themselves and with the exact solution (*Ex*) for the linear problem.

Let us define the maximal intensity of the reflected wave as

$$\begin{array}{c} \begin{array}{c} A_{Ref}\left(S\right)=\max_{t_{m}}\max_{z_{j}}\left|\right|A_{S}\left(t_{m},z_{j}\right){|}^{2}-|A_{Ex}\left(t_{m},z_{j}\right){|}^{2}|,0\leq j\leq N_{z},0\leq m\leq N_{t},\\ S=\{R,C,R\&C\}. \end{array} \end{array}$$ (87)

For definiteness, the computer simulation is made for the following parameters $L_{z}=20.0,L_{z_{c}}=15.0,D_{z}=0.0796,\phi =3.14,\chi =1$ and for the mesh step on z-coordinate *h* = 0.01.

#### The linear problem

**Fig 13:**
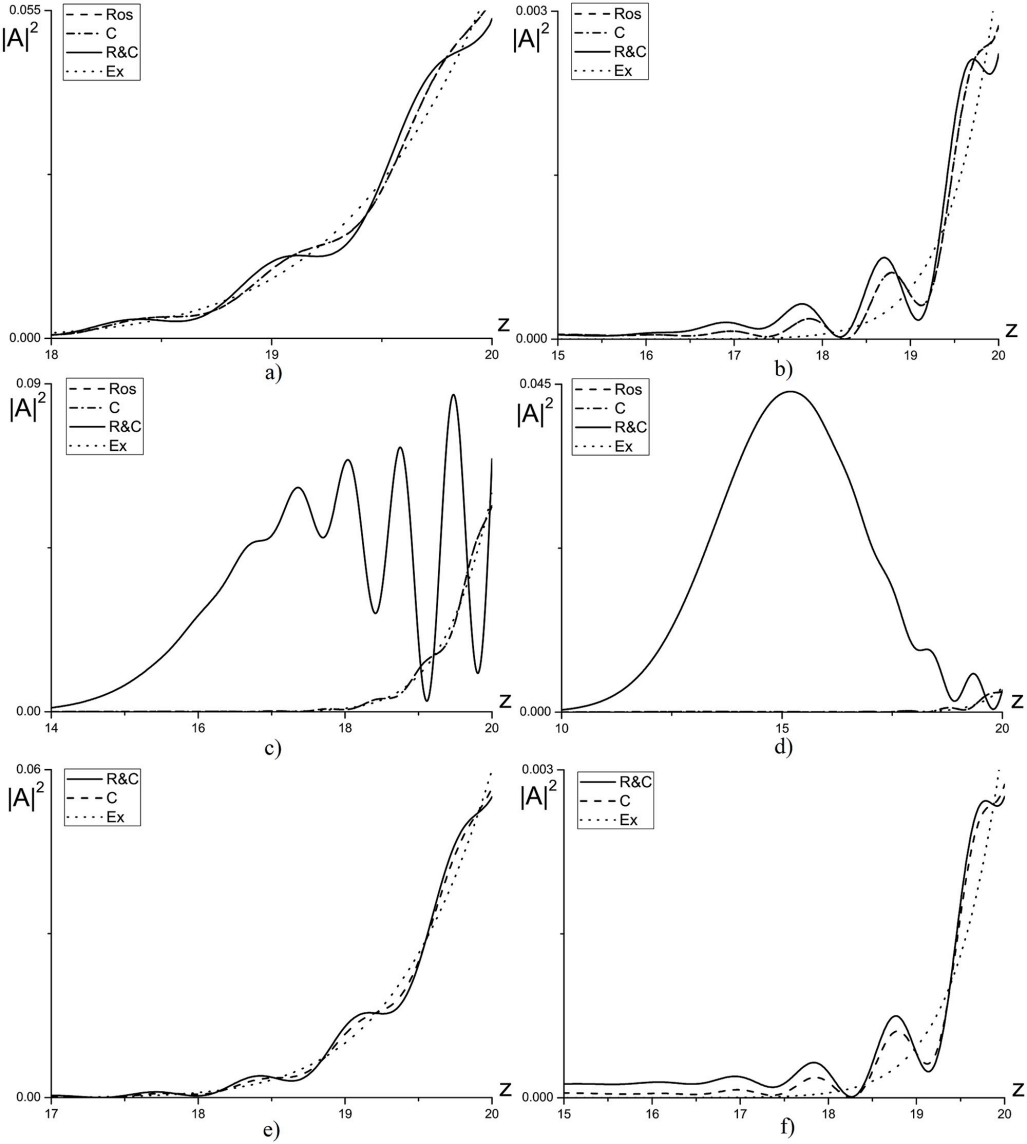
The intensity profile |*A*|^2^ computed at the time moments *t* = 7.5(*a*, *c*, *e*), 10(*b*, *d*, *f*) for the mesh steps *τ*_*R*_ = *τ* = 0.01(*a*, *b*, *c*, *d*); 0.001(*e*, *f*), and a number of nodes for the sub-domains: *N*_*R*_ = 100(*a*, *b*); 10(*c*, *d*, *e*, *f*).

In Fig 13 the intensity profiles, obtained using three methods mentioned above for computation of the laser pulse propagation in a linear medium (*ψ* = 0.0) are compared. As it follows from (Fig 13a, 13b, 13c and 13d) the difference between the numerical solution, obtained using combined method, and the corresponding solutions, obtained using other finite-difference schemes, strongly depends on the mesh nodes number (*N*_*R*_) in sub-domains and on the mesh steps *τ*, *τ*_*R*_. So, for the mesh step *τ*_*R*_ = *τ* = 0.01 and with decreasing the nodes number *N*_*R*_ from 100 to 10 the reflected wave significantly increases (see Fig 13c and 13d). However, with decreasing the time step to the value *τ*_*R*_ = *τ* = 0.001 at fixed number of nodes in the sub-domains *N*_*R*_ = 10, the reflected wave intensity decreases noticeably (see Fig 13e and 13f) and this intensity becomes practically the same one as the intensity computed using other finite-difference schemes.

**Fig 14:**
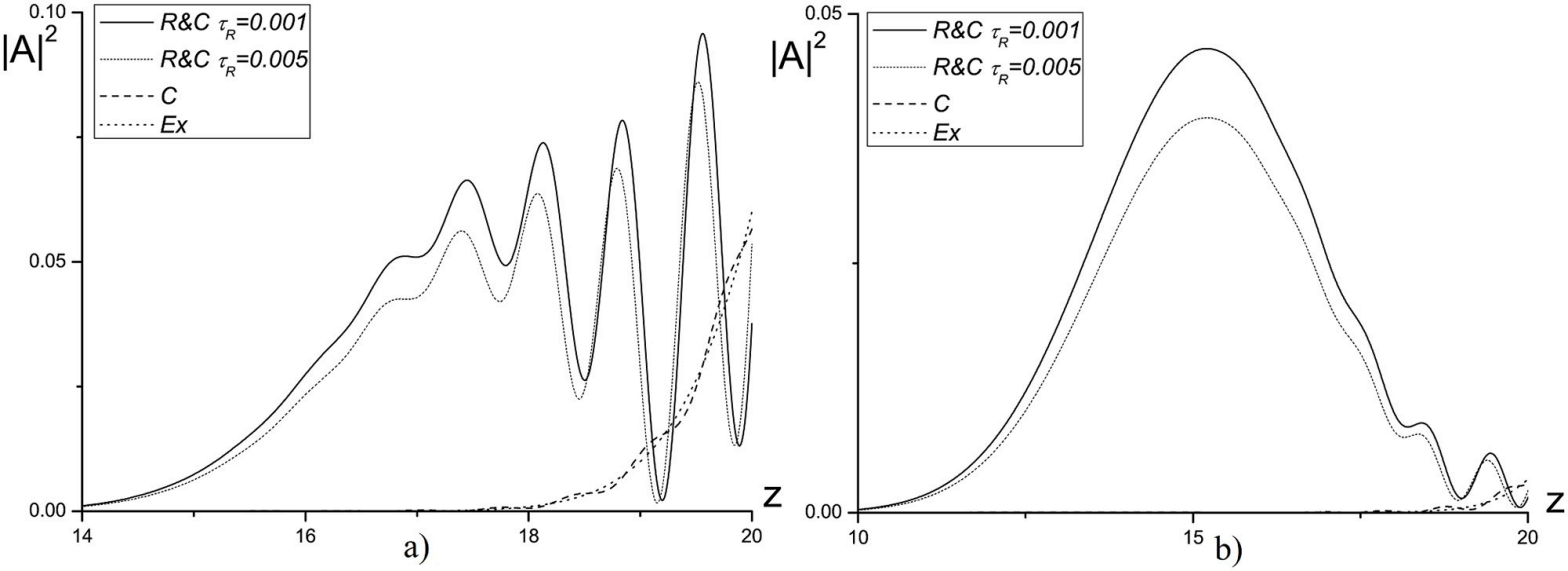
The intensity profile |*A*|^2^, computed at the time moments *t* = 7.5(*a*), 10(*b*) for the parameter *N*_*R*_ = 10 and the mesh steps *τ*_*R*_ = 0.001; 0.005, *τ* = 0.01.

To increase the effectiveness of the combined method we use decreasing time step for the problem solution in the sub-domains. In Fig 14 the numerical solutions, obtained using finite-difference scheme based on the combined method with the parameter *N*_*R*_ = 10 and the mesh steps *τ*_*R*_ = 0.001; 0.005, *τ* = 0.01, are presented. As it can be seen, decreasing the time step *τ*_*R*_ leads to weak decrease of the intensity for the wave reflected from the artificial boundary and does not provide a significant improvement of the computer simulation results.

**Fig 15:**
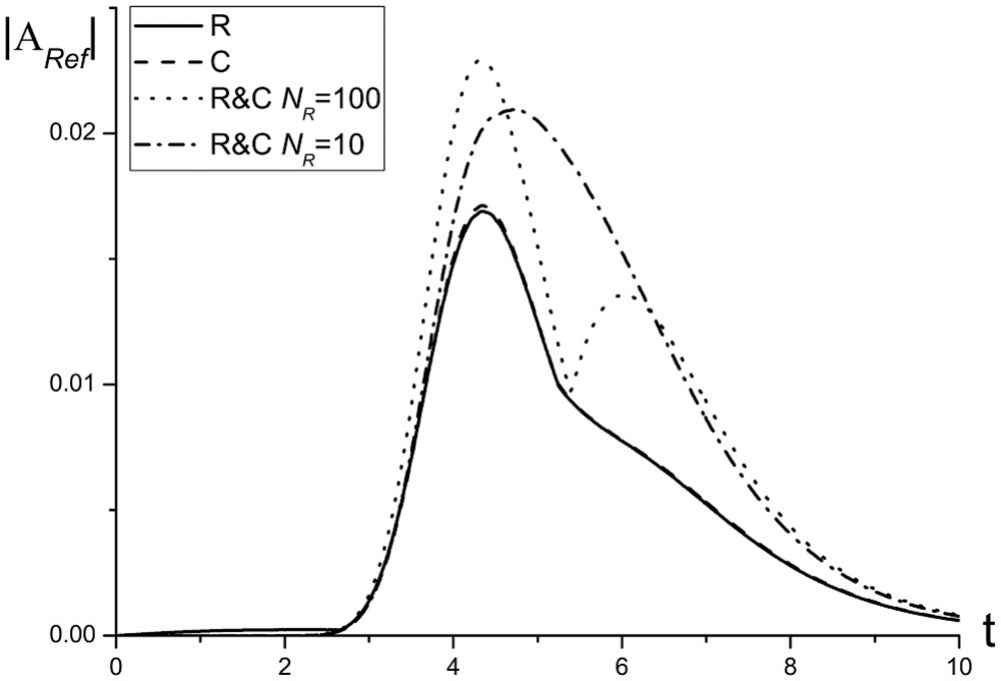
The evolution of reflected wave maximal amplitude in the time interval 0 ≤ *t* ≤ 10, computed using the finite-difference schemes *Ros*, *C* and *R*&*C* with *N*_*R*_ = 100 for the mesh step *τ* = 0.01 and *R*&*C* with *N*_*R*_ = 100 for the mesh step *τ* = 0.001.

A comparison between the maximal intensity of the reflected wave, obtained using the finite-difference scheme based on the combined method, and the corresponding intensities, obtained using other finite-difference schemes (see Fig 15), demonstrates, that use of the combined method leads to a bigger reflected wave amplitude even for the optimal parameters. However, this growth is not critical and doesn’t exceed the approximation error order.

**Fig 16:**
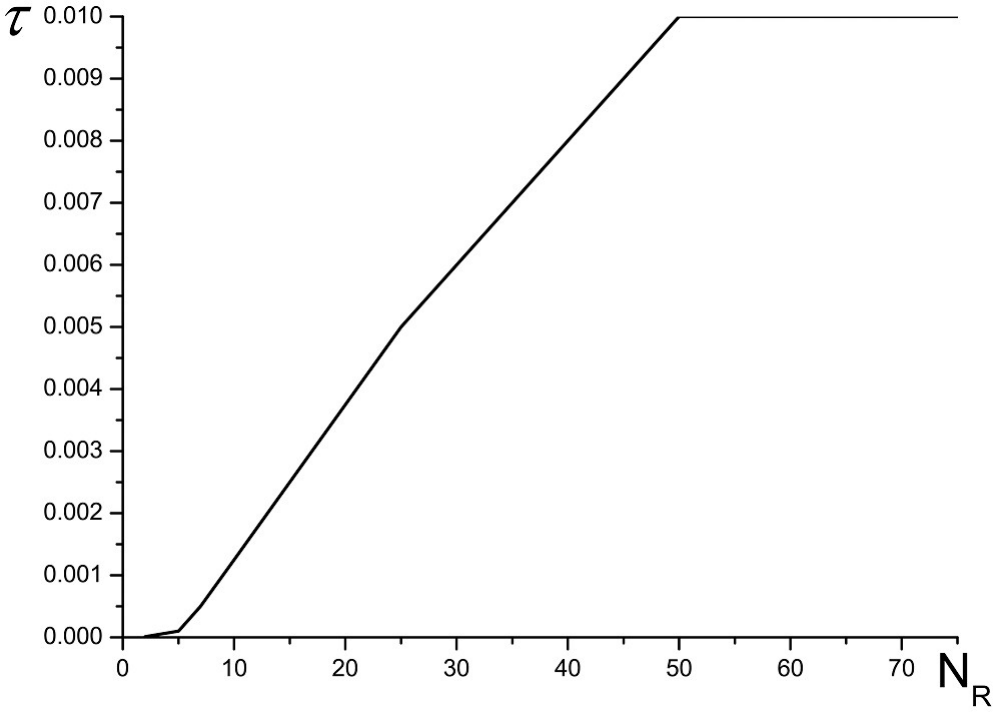
The time step dependence on a number of grid nodes in the sub-domain *N*_*R*_ to achieve by using the combined method the solution accuracy computed on the base of CFDS at the time moment *t* = 10.0.

It is important to compare the results, obtained using the finite-difference scheme based on the combined method and CFDS. In Fig 16 the dependence of mesh step on time, at which the results coincidence of computations for two finite-difference scheme is achieved, from the nodes number in the sub-domain *N*_*R*_ is depicted. Note that with decreasing the nodes number *N*_*R*_, it is necessary to decrease the time step to achieve the corresponding accuracy.

To estimate the effectiveness of proposed method, the comparison of the first invariant values deviation for three finite-difference schemes under consideration is demonstrated in Table 7. The results presented here are for the finite-difference scheme based on the Rosenbrock method and for the CFDS, computed with using the ABCs. As one can see, this deviation for the combined method is two times bigger then the corresponding value obtained for other finite-difference schemes. However, this values don’t exceed the theoretical estimations.

**Table 7:** The first invariant deviation computed using the finite-difference schemes under consideration for the parameters *L*_*z*_ = 20.0, *t* = 10.0 and the mesh steps *h* = *τ* = *τ*_*R*_ = 0.01.

Method	First invariant difference	Hamiltonian difference
*Ros*	0.000548643	0.0021649
*C*	0.000556224	0.0035698
*R*&*C*	0.001128941	0.0093432

#### Case of laser pulse self-focusing

**Fig 17:**
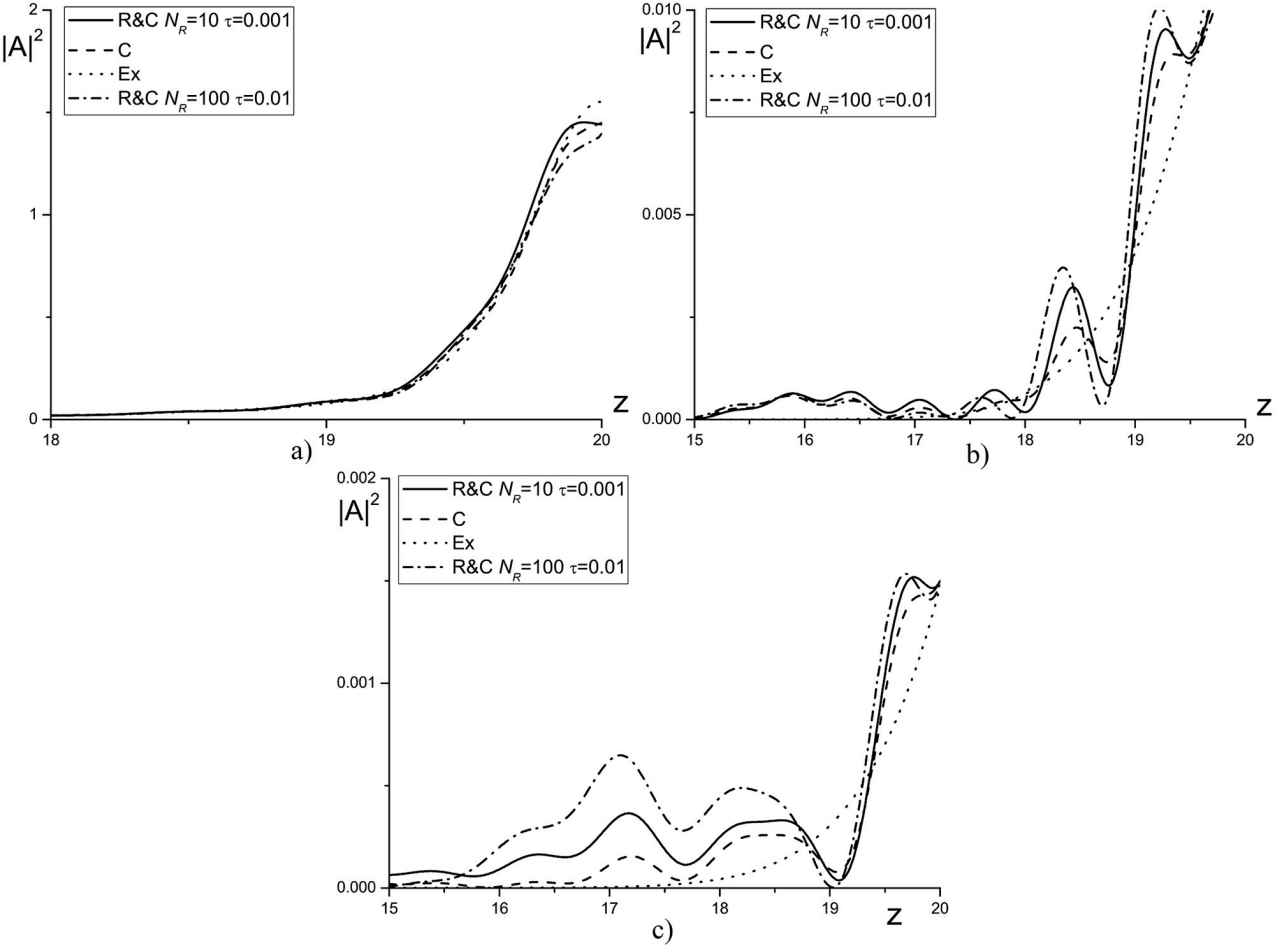
The intensity profile |*A*|^2^ computed at the time moments *t* = 5(*a*), 7.5(*b*), 10(*c*) for the parameter *ψ* = 1.0.

In Fig 17 the intensity profiles, computed using all finite-difference schemes for the nonlinear problem with *ψ* = 1.0, is shown. It should be stressed that in this case as an exact solution we use the numerical solution computed on the base of the CFDS with zero-value BCs in enough big domain with respect to the coordinate *z*.

As it can be seen in Fig 17a, the difference between the solution, obtained using the finite-difference scheme based on the combined method, and the exact solution has the value of about 0.1 in considered time moment. However, this difference does not preserve in time. On the other hand, the wave reflected from the artificial boundary is absent. Therefore, the difference in amplitude is due to the difference in velocity of wave transmitting through the boundary.

At the next considered time moments (see Fig 17b and 17c), the difference become significantly less and equals to 10^−3^ and 10^−4^, correspondingly. The maximal intensity of reflected wave doesn’t exceed the 10^−4^.

#### Case of laser pulse defocusing

**Fig 18:**
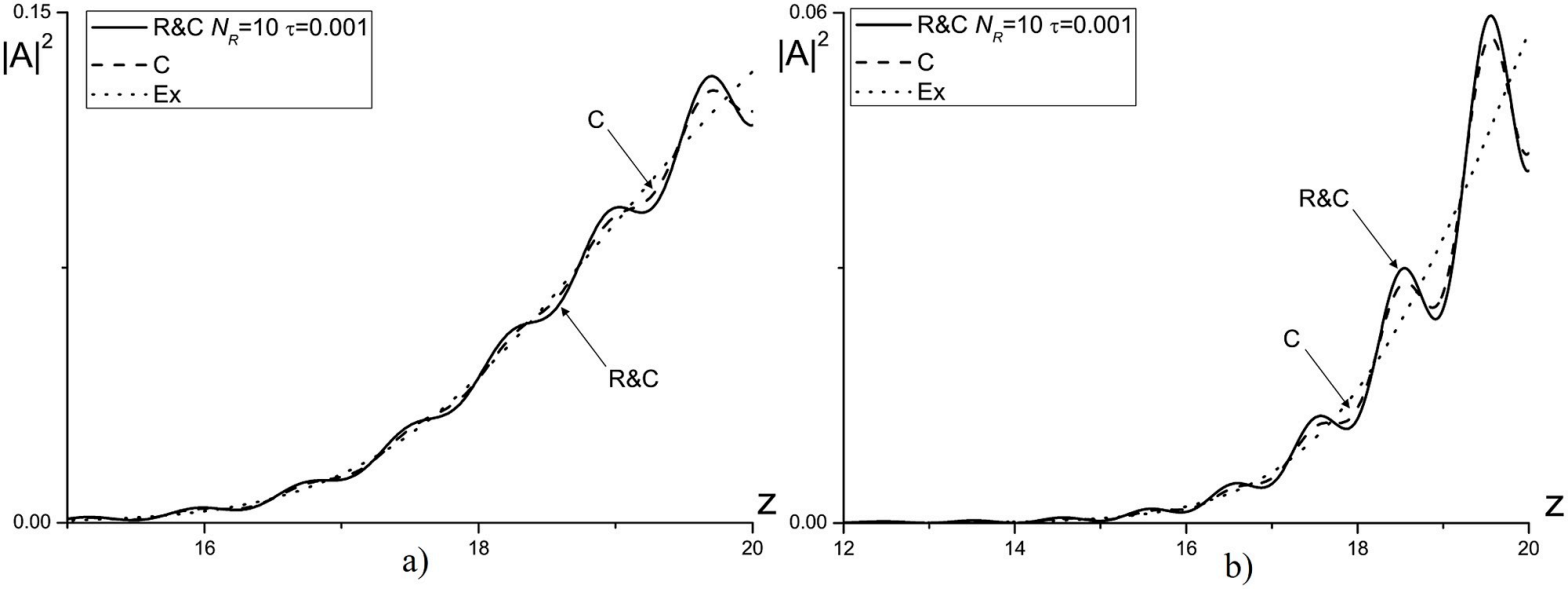
The intensity profile |*A*|^2^, computed at the time moments *t* = 7.5(*a*), 10(*b*) for the parameters *ψ* = −1.0, *N*_*R*_ = 10 and the mesh step *τ* = 0.001.

For the optical pulse propagation in defocusing medium the similar computation was made based on the Rosenbrock method, the CFDS and the finite-difference scheme for the combined method. In Fig 18 the intensity profiles, obtained using various finite-difference schemes, is shown for the parameters *ψ* = −1.0, *N*_*R*_ = 10, *τ* = *τ*_*R*_ = 0.001. Thus, the numerical solution obtained using the combined method coincides with the corresponding solution obtained using the CFDS. It should be stressed, that the maximal amplitude of the reflected wave shown in Fig 18 has the order 10^−2^ and it is worse than the corresponding result obtained for case of the pulse self-focusing case. This fact takes place because of the constant parameter *Ω*_*z*_. Moreover, the nonlinear distortion of the laser pulse at its front influences strongest on the phase wave front than at the laser pulse propagation in a self-focusing medium. Therefore, the optical radiation local wave number doesn’t coincide with the using *Ω*_*z*_ value for the ABCs.

In the conclusion of this section, we should note, that it is possible to arrange the computation of the combined method in a different way. We construct also the explicit finite-difference scheme on the basis of the predictor-corrector scheme instead of CFDS. Corresponding to this scheme, at the first stage we compute the solution on the upper time layer at using the Rosenbrock method in full domain under consideration. Then, at the second stage we compute the problem solution at using the predictor-corrector scheme: the nonlinear term in (34) is computed by using the solution obtained at previous stage with a help of the Rosenbrock method. The computer simulation results obtained using this explicit method are not shown in this paper, because of the solution accuracy, obtained using this scheme, is two or three times worse than the corresponding accuracy, achieved using the CFDS, Rosenbrock method and combined method for all cases, which considered above (linear, self-focusing and defocusing problem).

## 2D Schrödinger equation

### Problem statement and problem invariants

Let us consider the 2D nonlinear Schrödinger equation describing the femtosecond pulse propagation in 2D PC

$$\begin{array}{c} \begin{array}{c} \varepsilon \left(z,x\right)\frac{\partial A}{\partial t}+ıD_{z}\frac{\partial^{2}A}{\partial z^{2}}+ıD_{x}\frac{\partial^{2}A}{\partial x^{2}}{+ı\phi (\varepsilon \left(z,x\right)+\psi \left(z,x\right)|A|}^{2})A=0,\\ t>0,0<z<L_{z},0<x<L_{x} \end{array} \end{array}$$ (88)

with initial complex amplitude distribution

$$\begin{array}{c} {A|}_{t=0}=e^{-{\left(z-L_{z_{c}}\right)}^{2}/a_{z}^{2}-{\left(x-L_{x_{c}}\right)}^{2}/a_{x}^{2}+ı2\pi \chi \left(z-L_{z_{c}}\right)}, \end{array}$$ (89)

and ABCs are written in the following way

$$\begin{array}{c} \begin{array}{c} (\frac{\partial A}{\partial t}-2D_{z}\Omega_{z_{l}}\frac{\partial A}{\partial z}+ıD_{z}\Omega_{z_{l}}^{2}{A+ı\phi (1+\psi |A|}^{2})A){|}_{z=0}=0,\\ (\frac{\partial A}{\partial t}+2D_{z}\Omega_{z_{r}}\frac{\partial A}{\partial z}+ıD_{z}\Omega_{z_{r}}^{2}{A+ı\phi (1+\psi |A|}^{2})A){|}_{z=L_{z}}=0,\\ (\frac{\partial A}{\partial t}-2D_{x}\Omega_{x_{b}}\frac{\partial A}{\partial x}+ıD_{x}\Omega_{x_{b}}^{2}{A+ı\phi (1+\psi |A|}^{2})A){|}_{x=0}=0,\\ (\frac{\partial A}{\partial t}+2D_{x}\Omega_{x_{u}}\frac{\partial A}{\partial x}+ıD_{x}\Omega_{x_{u}}^{2}{A+ı\phi (1+\psi |A|}^{2})A){|}_{x=L_{x}}=0. \end{array} \end{array}$$ (90)

In addition to the notations, introduced above, let us note, that in (88) *A*(*t*, *z*, *x*) is a slowly varying amplitude in time only; *x* denote a spatial coordinate; *L*_*x*_ is its maximal value; $L_{x_{c}}$ is the coordinate of the laser beam center at initial time moment along x-coordinate; *ε*(*z*, *x*), *ψ*(*z*, *x*) are the functions of a dielectric permittivity and nonlinearity coefficient. Parameter *D*_*x*_ is the real coefficient describing the beam diffraction along x-coordinate. Variables *a*_*z*_, *a*_*x*_ denote the beam radii along the corresponding coordinates. Parameters $\Omega_{z_{l}},\Omega_{z_{r}},\Omega_{x_{b}},\Omega_{x_{u}}$ are the local wave numbers of the laser beam near the low and upper artificial boundary along x-coordinate, left and right artificial boundary along z-coordinate, correspondingly. For definiteness we denote $\Omega_{z_{l}}=\Omega_{z_{r}}=\Omega_{z}=2\pi \chi ,\Omega_{x_{b}}=\Omega_{x_{u}}=\Omega_{x}$. This way of the local wave number $\Omega_{x_{b}},\Omega_{x_{u}}$ definition is valid if the initial beam center along x-coordinate coincides with the domain center, i.e. $L_{x_{c}}=\frac{L_{x}}{2}$.

The problem (88)–(90) possesses two following invariants (conservation laws):

$$\begin{array}{c} \begin{array}{c} I_{1}\left(t\right)=\int_{0}^{L_{z}}\int_{0}^{L_{x}}\varepsilon \left(z,x\right){\left|A\right|}^{2}dzdx-\int_{0}^{t}[2D_{z}\int_{0}^{L_{x}}Im((\frac{\partial A}{\partial z}A^{*}){{|}_{z=L_{z}}+(\frac{\partial A^{*}}{\partial z}A)|}_{z=0})dx+\\ +2D_{x}\int_{0}^{L_{z}}Im((\frac{\partial A}{\partial x}A^{*}){{|}_{x=L_{x}}+(\frac{\partial A^{*}}{\partial x}A)|}_{x=0})dz]d\eta , \end{array} \end{array}$$ (91)

$$\begin{array}{c} \begin{array}{c} I_{3}\left(t\right)=\int_{0}^{L_{z}}\int_{0}^{L_{x}}(-D_{z}|\frac{\partial A}{\partial z}{|}^{2}-D_{x}|\frac{\partial A}{\partial x}{|}^{2}{+\phi (\varepsilon \left(z,x\right)+0.5\psi \left(z,x\right)|A|}^{2}{)|A|}^{2})dzdx+\\ +\int_{0}^{t}[2D_{z}\int_{0}^{L_{x}}Re((\frac{\partial A}{\partial z}\frac{\partial A^{*}}{\partial \eta }){{|}_{z=L_{z}}-(\frac{\partial A}{\partial z}\frac{\partial A^{*}}{\partial \eta })|}_{z=0})dx+\\ +2D_{x}\int_{0}^{L_{z}}Re((\frac{\partial A}{\partial x}\frac{\partial A^{*}}{\partial \eta }){{|}_{x=L_{x}}-(\frac{\partial A}{\partial x}\frac{\partial A^{*}}{\partial \eta })|}_{x=0})dz]d\eta . \end{array} \end{array}$$ (92)

### CFDS construction for 2D case

Let us introduce an uniform grid *ω*

$$\begin{array}{c} \begin{array}{c} \omega =\omega_{t}\times \omega_{z}\times \omega_{x},\omega_{t}=\left\{t_{m}=m\tau ,m=0\ldots N_{t},h=\frac{L_{t}}{N_{t}}\right\},\omega_{z}=\{z_{j}=jh_{z},\\ j=0\ldots N_{z},h_{z}=\frac{L_{z}}{N_{z}}\},\omega_{x}=\left\{x_{k}=kh_{x},k=0\ldots N_{x},h_{x}=\frac{L_{x}}{N_{x}}\right\}. \end{array} \end{array}$$ (93)

**Fig 19:**
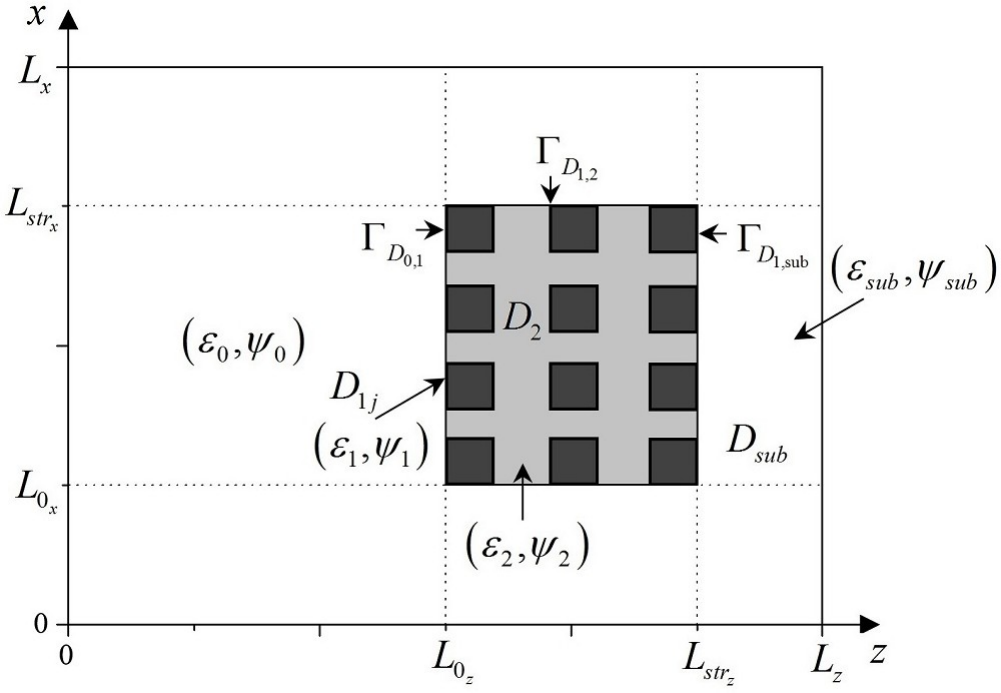
Dielectric permittivity and nonlinearity coefficients of the PC.

The complex amplitudes on the grid *ω* are defined as

$$\begin{array}{c} \begin{array}{c} A=A_{j,k}=A_{m,j,k}=A\left(t_{m},z_{j},x_{k}\right),\hat{A}={\hat{A}}_{j,k}={\hat{A}}_{m+1,j,k}=\\ =A\left(t_{m}+\tau ,z_{j},x_{k}\right),\overset{0.5}{A}=0.5\left(\hat{A}+A\right),|\overset{0.5}{A}{|}^{2}=0.5(|\hat{A}{|}^{2}+{\left|A\right|}^{2}). \end{array} \end{array}$$ (94)

$$\begin{array}{c} \left(\varepsilon ,\psi \right)=\{\begin{array}{cc} (\varepsilon_{0},\psi_{0}), & \left(z,x\right)\notin D_{1j},D_{2},D_{sub},\Gamma ;j=\bar{1,N_{str_{x}}\cdot N_{str_{z}}},\\ (\varepsilon_{1},\psi_{1}), & \left(z,x\right)\in D_{1j},j=\bar{1,N_{str_{x}}\cdot N_{str_{z}}}\\ (\frac{\varepsilon_{0}+\varepsilon_{1}}{2},\frac{\psi_{0}+\psi_{1}}{2}), & \left(z,x\right)\in \Gamma_{D_{0,1}},\\ (\frac{\varepsilon_{1}+\varepsilon_{2}}{2},\frac{\psi_{1}+\psi_{2}}{2}), & \left(z,x\right)\in \Gamma_{D_{1,2}},\\ (\frac{\varepsilon_{1}+\varepsilon_{sub}}{2},\frac{\psi_{1}+\psi_{sub}}{2}), & \left(z,x\right)\in \Gamma_{D_{1,sub}},\\ (\varepsilon_{2},\psi_{2}), & \left(z,x\right)\in D_{2},\\ (\frac{\varepsilon_{0}+\varepsilon_{2}}{2},\frac{\psi_{0}+\psi_{2}}{2}), & \left(z,x\right)\in \Gamma_{D_{0,2}},\\ (\frac{\varepsilon_{2}+\varepsilon_{sub}}{2},\frac{\psi_{2}+\psi_{sub}}{2}), & \left(z,x\right)\in \Gamma_{D_{2,sub}},\\ (\varepsilon_{sub},\psi_{sub}), & \left(z,x\right)\in D_{sub},\\ (\frac{\varepsilon_{0}+\varepsilon_{sub}}{2},\frac{\psi_{0}+\psi_{sub}}{2}), & \left(z,x\right)\in \Gamma_{D_{0,sub}},\\ (\frac{\varepsilon_{p}+\varepsilon_{q}+\varepsilon_{l}+\varepsilon_{k}}{4},\frac{\psi_{p}+\psi_{q}+\psi_{l}+\psi_{k}}{2}), & \left(z,x\right)\in \Gamma_{D_{p,q}}\cap \Gamma_{D_{l,k}},p,q,l,k\in \left\{0,1,2,sub\right\}, \end{array} \end{array}$$ (95)

where $D_{1j},D_{2},j=\bar{1,N_{str_{x}}\cdot N_{str_{z}}},\varepsilon_{1},\varepsilon_{2}$ and *ψ*_1_, *ψ*_2_ are domains, the dielectric permittivity and nonlinearity coefficient of PC’s layers, correspondingly, *ε*_*sub*_, *ψ*_*sub*_ is a dielectric permittivity and nonlinearity coefficient of a substrate, which is located behind the PC; *ε*_0_, *ψ*_0_ is a dielectric permittivity and nonlinearity coefficient of the medium before the PC; $N_{str_{x}},N_{str_{z}}$ is a number of structure elements along x and z coordinates, correspondingly. $\left(L_{0_{x}},L_{0_{z}}\right)$ is dimensionless coordinate of the PC’s face, $L_{str_{x}},L_{str_{z}}$ defines the longitudinal size of the PC along x and z coordinates. Γ = Γ_0,1_ ∪ Γ_0,2_ ∪ Γ_0,*sub*_ ∪ Γ_1,2_ ∪ Γ_1,*sub*_ ∪ Γ_2,*sub*_ are the boundaries between two domains with different dielectric permittivity and nonlinearity coefficient. We should note that the index *sub* in the boundaries definition corresponds to the substrate. For convenience, the introduced parameters and arrangement of the PC are depicted on Fig 19.

For the problem under consideration (88)–(90), we develop the following finite-difference scheme:

$$\begin{array}{c} \begin{array}{c} \varepsilon \frac{\hat{A}-A}{\tau }+ıD_{z}\Lambda_{\bar{z}z}\overset{0.5}{A}+ıD_{x}\Lambda_{\bar{x}x}\overset{0.5}{A}+ı\phi (\varepsilon +\psi |\overset{0.5}{A}{|}^{2})\overset{0.5}{A}=0,j=\bar{1,N_{z}-1},\\ k=\bar{1,N_{x}-1},m=\bar{0,N_{t}-1}, \end{array} \end{array}$$ (96)

with initial conditions

$$\begin{array}{c} {A|}_{t=0}=e^{-{\left(jh_{z}-L_{z_{c}}\right)}^{2}/a_{z}^{2}-{\left(kh_{x}-L_{x_{c}}\right)}^{2}/a_{x}^{2}+ı2\pi \chi \left(jh_{z}-L_{z_{c}}\right)} \end{array}$$ (97)

and ABCs

$$\begin{array}{c} \begin{array}{c} \frac{{\hat{A}}_{0,k}-A_{0,k}}{\tau }-2D_{z}\Omega_{z}\frac{{\overset{0.5}{A}}_{1,k}-{\overset{0.5}{A}}_{0,k}}{h_{z}}+ıD_{z}\Omega_{z}^{2}{\overset{0.5}{A}}_{0,k}+ı\phi (1+\psi |{\overset{0.5}{A}}_{0,k}{|}^{2}){\overset{0.5}{A}}_{0,k}=0,\\ \frac{{\hat{A}}_{N_{z},k}-A_{N_{z},k}}{\tau }+2D_{z}\Omega_{z}\frac{{\overset{0.5}{A}}_{N_{z},k}-{\overset{0.5}{A}}_{N_{z}-1,k}}{h_{z}}+ıD_{z}\Omega_{z}^{2}{\overset{0.5}{A}}_{N_{z},k}+ı\phi (1+\\ +\psi |{\overset{0.5}{A}}_{N_{z},k}{|}^{2}){\overset{0.5}{A}}_{N_{z},k}=0,\;k=\bar{1,N_{x}},m=\bar{0,N_{t}-1},\\ \frac{{\hat{A}}_{j,0}-A_{j,0}}{\tau }-2D_{x}\Omega_{x}\frac{{\overset{0.5}{A}}_{j,1}-{\overset{0.5}{A}}_{j,0}}{h_{x}}+ıD_{x}\Omega_{x}^{2}{\overset{0.5}{A}}_{j,0}+ı\phi (1+\psi |{\overset{0.5}{A}}_{j,0}{|}^{2}){\overset{0.5}{A}}_{j,0}=0,\\ \frac{{\hat{A}}_{j,N_{x}}-A_{j,N_{x}}}{\tau }+2D_{x}\Omega_{x}\frac{{\overset{0.5}{A}}_{j,N_{x}}-{\overset{0.5}{A}}_{j,N_{x}-1}}{h_{z}}+ıD_{x}\Omega_{x}^{2}{\overset{0.5}{A}}_{j,N_{x}}+ı\phi (1+\\ +\psi |{\overset{0.5}{A}}_{j,N_{x}}{|}^{2}){\overset{0.5}{A}}_{j,N_{x}}=0,\;j=\bar{1,N_{z}},m=\bar{0,N_{t}-1}. \end{array} \end{array}$$ (98)

The 2D problem under consideration is nonlinear one, therefore for its computation we use a two-stage iteration process [52, 53]. The first stage of the iteration process is written in the following form:

$$\begin{array}{c} \begin{array}{c} \varepsilon \frac{\overset{s+1}{\hat{A}}-A}{\tau }+ıD_{z}\Lambda_{\bar{z}z}\overset{s+1}{\overset{0.5}{A}}+ıD_{x}\Lambda_{\bar{x}x}\overset{s}{\overset{0.5}{A}}+ı\phi (\varepsilon +\psi |\overset{s}{\overset{0.5}{A}}{|}^{2})\overset{s}{\overset{0.5}{A}}=0,\\ j=\bar{1,N_{z}-1},k=\bar{1,N_{x}-1},m=\bar{0,N_{t}-1}, \end{array} \end{array}$$ (99)

$$\begin{array}{c} \begin{array}{c} \frac{\overset{s+1}{{\hat{A}}_{0,k}}-A_{0,k}}{\tau }-2D_{z}\Omega_{z}\frac{\overset{s+1}{\overset{0.5}{A_{1,k}}}-\overset{s+1}{\overset{0.5}{A_{0,k}}}}{h_{z}}+ıD_{z}\Omega_{z}^{2}\overset{s+1}{\overset{0.5}{A_{0,k}}}+ı\phi (1+\psi |\overset{s}{\overset{0.5}{A_{0,k}}}{|}^{2})\overset{s}{\overset{0.5}{A_{0,k}}}=0,\\ \frac{\overset{s+1}{{\hat{A}}_{N_{z},k}}-A_{N_{z},k}}{\tau }+2D_{z}\Omega_{z}\frac{\overset{s+1}{\overset{0.5}{A_{N_{z},k}}}-\overset{s+1}{\overset{0.5}{A_{N_{z}-1,k}}}}{h_{z}}+ıD_{z}\Omega_{z}^{2}\overset{s+1}{\overset{0.5}{A_{N_{z},k}}}+\\ +ı\phi (1+\psi |\overset{s}{\overset{0.5}{A_{N_{z},k}}}{|}^{2})\overset{s}{\overset{0.5}{A_{N_{z},k}}}=0,\;k=\bar{1,N_{x}},m=\bar{0,N_{t}-1} \end{array} \end{array}$$ (100)

and corresponds to a solution of the difference problem (96)–(98) along z-coordinate.

The second stage of the iteration process is written in the way:

$$\begin{array}{c} \begin{array}{c} \varepsilon \frac{\overset{s+2}{\hat{A}}-A}{\tau }+ıD_{z}\Lambda_{\bar{z}z}\overset{s+1}{\overset{0.5}{A}}+ıD_{x}\Lambda_{\bar{x}x}\overset{s+2}{\overset{0.5}{A}}+ı\phi (\varepsilon +\psi |\overset{s}{\overset{0.5}{A}}{|}^{2})\overset{s}{\overset{0.5}{A}}=0,\\ j=\bar{1,N_{z}-1},k=\bar{1,N_{x}-1},m=\bar{0,N_{t}-1}, \end{array} \end{array}$$ (101)

$$\begin{array}{c} \begin{array}{c} \frac{\overset{s+2}{{\hat{A}}_{j,0}}-A_{j,0}}{\tau }-2D_{x}\Omega_{x}\frac{\overset{s+2}{\overset{0.5}{A_{j,1}}}-\overset{s+2}{\overset{0.5}{A_{j,0}}}}{h_{x}}+ıD_{x}\Omega_{x}^{2}\overset{s+2}{\overset{0.5}{A_{j,0}}}+ı\phi (1+\psi |\overset{s}{\overset{0.5}{A_{j,0}}}{|}^{2})\overset{s}{\overset{0.5}{A_{j,0}}}=0,\\ \frac{\overset{s+2}{{\hat{A}}_{j,N_{x}}}-A_{j,N_{x}}}{\tau }+2D_{x}\Omega_{x}\frac{\overset{s+2}{\overset{0.5}{A_{j,N_{x}}}}-\overset{s+2}{\overset{0.5}{A_{j,N_{x}-1}}}}{h_{x}}+ıD_{x}\Omega_{x}^{2}\overset{s+2}{\overset{0.5}{A_{j,N_{x}}}}+\\ +ı\phi (1+\psi |\overset{s}{\overset{0.5}{A_{j,N_{x}}}}{|}^{2})\overset{s}{\overset{0.5}{A_{j,N_{x}}}}=0,\;j=\bar{1,N_{z}},m=\bar{0,N_{t}-1} \end{array} \end{array}$$ (102)

and corresponds to a solution of the difference problem (96)–(98) along x-coordinate.

The mesh function on the upper time layer at zero iteration (*s* = 0) is chosen as it presented in (39). The iteration process is stopped if the following condition is valid

$$\begin{array}{c} \underset{z_{j},x_{k}}{max}|\overset{s+2}{\hat{A}}-\overset{s}{\hat{A}}|\leq {\tilde{\theta }}_{1}\underset{z_{j},x_{k}}{max}\left|\overset{s}{\hat{A}}\right|+{\tilde{\theta }}_{2}, \end{array}$$ (103)

where ${\tilde{\theta }}_{1},{\tilde{\theta }}_{2}>0$ are the real constants.

### Finite-difference scheme based on the Rosenbrock method for 2D case

Let us define the grid functions in the grid *ω*, which are defined in (93)

$$\begin{array}{c} \begin{array}{c} {\bar{A}}_{m,j,k}={\bar{A}}_{h}\left(t_{m},z_{j},x_{k}\right)={\bar{u}}_{m,j,k}+ı{\bar{v}}_{m,j,k},{\bar{u}}_{m,j,k}={\bar{u}}_{h}\left(t_{m},z_{j},x_{k}\right),\\ {\bar{v}}_{m,j,k}={\bar{v}}_{h}\left(t_{m},z_{j},x_{k}\right),\;0\leq j\leq N_{z},0\leq k\leq N_{x},0\leq m\leq N_{t} \end{array} \end{array}$$ (104)

and the vectors

$$\begin{array}{c} \begin{array}{c} \hat{U}=(u_{m+1,0,0},u_{m+1,0,1},\ldots ,u_{m+1,0,N_{x}},u_{m+1,1,0},\ldots ,u_{m+1,1,N_{x}},\ldots ,\\ u_{m+1,N_{z},0},\ldots ,u_{m+1,N_{z},N_{x}},v_{m+1,0,0},\ldots ,v_{m+1,N_{z},N_{x}}),\\ U=(u_{m,0,0},u_{m,0,1},\ldots ,u_{m,0,N_{x}},u_{m,1,0},\ldots ,u_{m,1,N_{x}},\ldots ,\\ u_{m,N_{z},0},\ldots ,u_{m,N_{z},N_{x}},v_{m,0,0},v_{m,0,1},\ldots ,v_{m,N_{z},N_{x}}),m=\bar{0,N_{t}-1} \end{array} \end{array}$$ (105)

According to the Rosenbrock method, the difference solution on the next time layer is computed as it is written in (18). The vector $\bar{k}$, which is a solution of the linear equations, in this case is written in the following form

$$\begin{array}{c} \begin{array}{cc} \left(E-\beta \tau G_{U}\right)\bar{k}=G\left(U\right), & \;j=\bar{1,N_{z}-1},k=\bar{1,N_{x}-1},\\ \left(E-\beta \tau G_{U_{b_{z}}}\right)\bar{k}=G\left(U_{b_{z}}\right), & \;j=0,N_{z},k=\bar{1,N_{x}-1},\\ \left(E-\beta \tau G_{U_{b_{x}}}\right)\bar{k}=G\left(U_{b_{x}}\right), & \;j=\bar{1,N_{z}-1},k=0,N_{x},m=\bar{0,N_{t}-1}. \end{array} \end{array}$$ (106)

The vectors $G\left(U\right),G\left(U_{b_{z}}\right),G\left(U_{b_{x}}\right)$ are defined as

$$\begin{array}{c} \begin{array}{c} G(\bar{U})=(\begin{array}{c} \frac{D_{z}}{\varepsilon }\Lambda_{\bar{z}z}{\bar{v}}_{j,k}+\frac{D_{x}}{\varepsilon }\Lambda_{\bar{x}x}{\bar{v}}_{j,k}+\phi \left(1+\frac{\psi }{\varepsilon }\left({\bar{u}}_{j,k}^{2}+{\bar{v}}_{j,k}^{2}\right)\right){\bar{v}}_{j,k}\\ -\frac{D_{z}}{\varepsilon }\Lambda_{\bar{z}z}{\bar{u}}_{j,k}-\frac{D_{x}}{\varepsilon }\Lambda_{\bar{x}x}{\bar{u}}_{j,k}-\phi \left(1+\frac{\psi }{\varepsilon }\left({\bar{u}}_{j,k}^{2}+{\bar{v}}_{j,k}^{2}\right)\right){\bar{u}}_{j,k}, \end{array}),\\ j=\bar{1,N_{z}-1},\;k=\bar{1,N_{x}-1},m=\bar{0,N_{t}-1}, \end{array} \end{array}$$ (107)

$$\begin{array}{c} \begin{array}{c} G(U_{b_{z}})=(\begin{array}{c} 2D_{z}\Omega_{z}\frac{u_{1,k}-u_{0,k}}{h_{z}}+\left(D_{z}\Omega_{z}^{2}+\phi \left(1+\psi \left(u_{0,k}^{2}+v_{0,k}^{2}\right)\right)\right)v_{0,k}\\ 2D_{z}\Omega_{z}\frac{v_{1,k}-v_{0,k}}{h_{z}}-\left(D_{z}\Omega_{z}^{2}+\phi \left(1+\psi \left(u_{0,k}^{2}+v_{0,k}^{2}\right)\right)\right)u_{0,k}\\ -2D_{z}\Omega_{z}\frac{u_{N_{z},k}-u_{N_{z}-1,k}}{h_{z}}+\left(D_{z}\Omega_{z}^{2}+\phi \left(1+\psi \left(u_{N_{z},k}^{2}+v_{N_{z},k}^{2}\right)\right)\right)v_{N_{z},k}\\ -2D_{z}\Omega_{z}\frac{v_{N_{z},k}-v_{N_{z}-1,k}}{h_{z}}-\left(D_{z}\Omega_{z}^{2}+\phi \left(1+\psi \left(u_{N_{z},k}^{2}+v_{N_{z},k}^{2}\right)\right)\right)u_{N_{z},k} \end{array}),\\ k=\bar{1,N_{x}-1},m=\bar{0,N_{t}-1}, \end{array} \end{array}$$ (108)

$$\begin{array}{c} \begin{array}{c} G(U_{b_{x}})=(\begin{array}{c} 2D_{x}\Omega_{x}\frac{u_{j,1}-u_{j,0}}{h_{x}}+\left(D_{x}\Omega_{x}^{2}+\phi \left(1+\psi \left(u_{j,0}^{2}+v_{j,0}^{2}\right)\right)\right)v_{j,0}\\ 2D_{x}\Omega_{x}\frac{v_{j,1}-v_{j,0}}{h_{x}}-\left(D_{x}\Omega_{x}^{2}+\phi \left(1+\psi \left(u_{j,0}^{2}+v_{j,0}^{2}\right)\right)\right)u_{j,0}\\ -2D_{x}\Omega_{x}\frac{u_{j,N_{x}}-u_{j,N_{x}-1}}{h_{x}}+\left(D_{x}\Omega_{x}^{2}+\phi \left(1+\psi \left(u_{j,N_{x}}^{2}+v_{j,N_{x}}^{2}\right)\right)\right)v_{j,N_{x}}\\ -2D_{x}\Omega_{x}\frac{v_{j,N_{x}}-v_{j,N_{x}-1}}{h_{x}}-\left(D_{x}\Omega_{x}^{2}+\phi \left(1+\psi \left(u_{j,N_{x}}^{2}+v_{j,N_{x}}^{2}\right)\right)\right)u_{j,N_{x}} \end{array}),\\ j=\bar{1,N_{z}-1},m=\bar{0,N_{t}-1}. \end{array} \end{array}$$ (109)

Above *G*_*U*_ is the Jacobian for the vector *G*(*U*) with respect to the mesh function *U* (105), $G_{U_{b_{z}}},G_{U_{b_{x}}}$ are the corresponding Jacobians of the right part vectors $G\left(U_{b_{z}}\right),G\left(U_{b_{x}}\right)$, which corresponds to the ABCs. The way of the Jacobians computation is presented above for the 1D problem in (27) and (28).

As mentioned above, the equation set (106) is solved by using the Thomas algorithm. Obviously, in the case under consideration, the matrices dimension as well as the vectors dimension increases many times. For example, a dimension of the vector $\bar{k}$ for the 2D problem is equal to 2(*N*_*x*_ × *N*_*z*_) and the matrix (*E* − *τβG*_*U*_) dimension is equal to (2(*N*_*x*_ × *N*_*z*_))^2^, correspondingly. Therefore, a dimension of the vector *Y*_*j*_ is equal to 4*N*_*x*_ and the matrices *Q*, *C*_*j*_, *B* dimension is equal to (4*N*_*x*_)^2^.

### Combined method for 2D case

**Fig 20:**
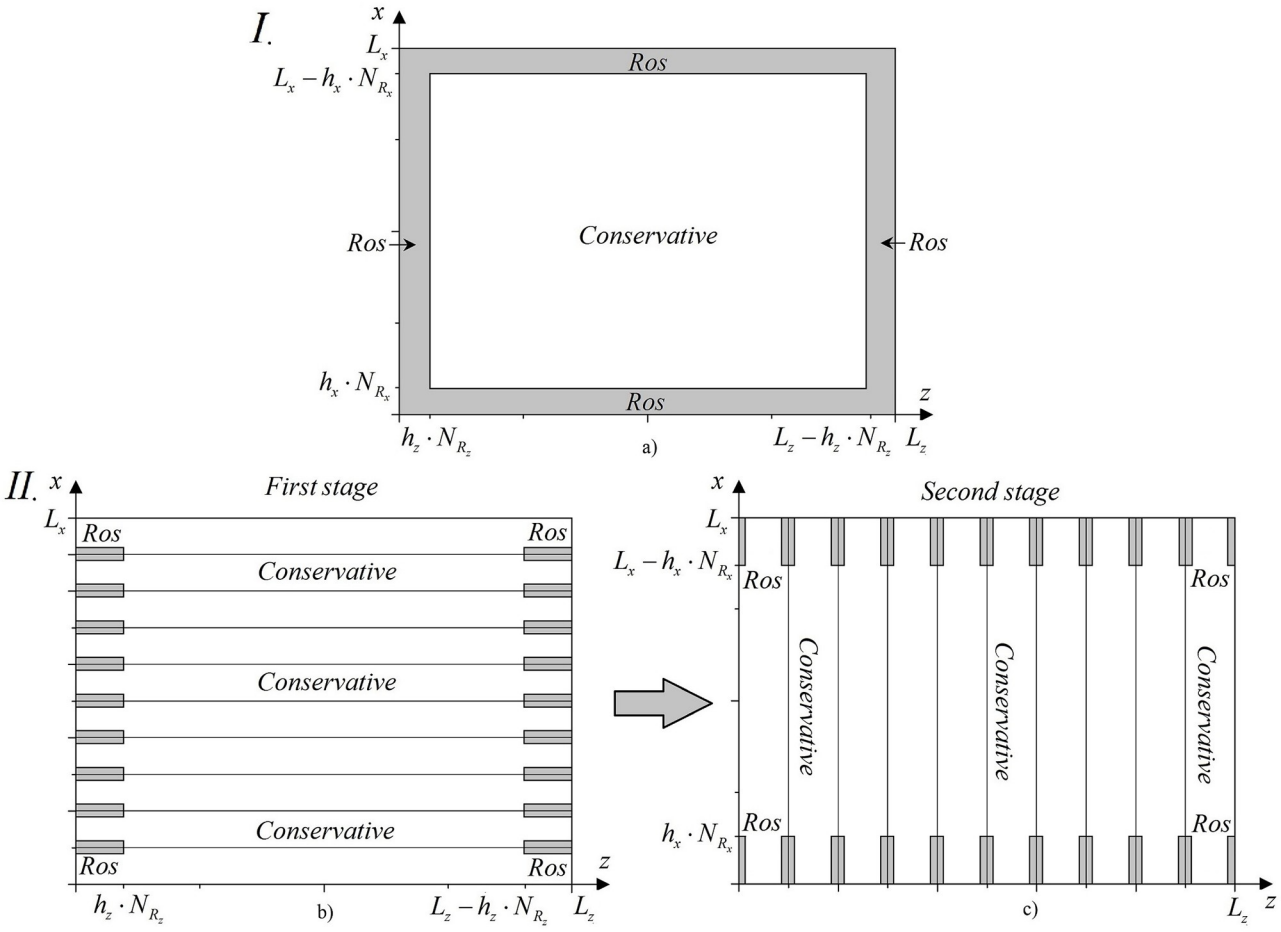
Two ways for computation using the combined method in 2D case.

The combined method in 2D case can be realized in two different ways. The first one consists in using the Rosenbrock method near the boundaries with ABCs (see Fig 20a). Then, CFDS is used in full domain with the BCs, which are computed by using the Rosenbrock method (it means that we solve the Dirichlet problem).

The second one consist in a reduction of the 2D difference problem to a sequence of the 1D difference problem. Below we consider in detail the construction of the combined method at using the second way because in this case the dimensions of the matrices are essentially less than at using the Rosenbrock method for the 2D problem solution directly.

We start from the difference Eqs (99)–(102) with the ABC. However, we modify this iteration process in accordance with the definition of the combined method for the 1D nonlinear Schrödinger equation. At each of these stages we use the Dirichlet BCs instead of (100), (102) and they are computed by using the Rosenbrock method in the domain near the artificial boundaries. Then, we apply the CFDS for the problem solution (see Fig 20b and 20c). Obviously, at this stage of the problem solution we use the Dirichlet BCs.

Thus, let’s consider the construction of the combined method. At first, we compute the problem solution in the grid nodes near the boundaries at using Rosenbrock method with corresponding ABCs (90). With this aim we introduce the sub-domains. For example, let’s start from a direction along z-coordinate. We denote a number of the grid nodes in sub-domains along z-coordinate as $N_{z_{R}}$ and write the finite-difference scheme, based on the Rosenbrock method in these sub-domains:

$$\begin{array}{c} {\hat{A}}_{Ros}=A+\tau_{R}Re\bar{q},\;j=\bar{0,N_{z_{R}}};\bar{N_{z}-N_{z_{R}},N_{z}},k=\bar{1,N_{x}-1},m=\bar{0,N_{t}-1}. \end{array}$$ (110)

where *τ*_*R*_ is a time step used for a computation in the sub-domains of the problem solution, $Re\bar{q}$ is a real part of the solution of the linear equations:

$$\begin{array}{c} \begin{array}{cc} \left(E-\beta \tau_{R}G_{U}\right)\bar{q}=G\left(U\right), & \;j=\bar{1,N_{z_{R}}};\bar{N_{z}-N_{z_{R}},N_{z}-1},\\ \left(E-\beta \tau_{R}G_{U_{b}}\right)\bar{q}=G\left(U_{b}\right), & \;j=0,N_{z},k=\bar{1,N_{x}-1},m=\bar{0,N_{t}-1}. \end{array} \end{array}$$ (111)

A parameter *β* of the Rosenbrock method, is chosen as *β* = 0.5. Matrix *G*_*U*_ is the Jacobian for the vector *G*(*U*)

$$\begin{array}{c} \begin{array}{c} G(U)=(\begin{array}{c} \frac{D_{z}}{\varepsilon }\Lambda_{\bar{z}z}v_{j,k}+\frac{D_{x}}{\varepsilon }\Lambda_{\bar{x}x}v_{j,k}+\phi \left(1+\frac{\psi }{\varepsilon }\left(u_{j,k}^{2}+v_{j,k}^{2}\right)\right)v_{j,k}\\ -\frac{D_{z}}{\varepsilon }\Lambda_{\bar{z}z}u_{j,k}-\frac{D_{x}}{\varepsilon }\Lambda_{\bar{x}x}u_{j,k}-\phi \left(1+\frac{\psi }{\varepsilon }\left(u_{j,k}^{2}+v_{j,k}^{2}\right)\right)u_{j,k}, \end{array}),\\ j=\bar{1,N_{z_{R}}},\bar{N_{z}-N_{z_{R}},N_{z}-1},k=\bar{1,N_{x}-1},m=\bar{0,N_{t}-1}. \end{array} \end{array}$$ (112)

and $G_{U_{b}}$ is the Jacobian of the right part vector *G*(*U*_*b*_), which corresponds to the ABCs and is written in the following form

$$\begin{array}{c} \begin{array}{c} G(U_{b})=(\begin{array}{c} 2D_{z}\Omega_{z}\frac{u_{1,k}-u_{0,k}}{h_{z}}+\left(D_{z}\Omega_{z}^{2}+\phi \left(1+\psi \left(u_{0,k}^{2}+v_{0,k}^{2}\right)\right)\right)v_{0,k}\\ 2D_{z}\Omega_{z}\frac{v_{1,k}-v_{0,k}}{h_{z}}-\left(D_{z}\Omega_{z}^{2}+\phi \left(1+\psi \left(u_{0,k}^{2}+v_{0,k}^{2}\right)\right)\right)u_{0,k}\\ -2D_{z}\Omega_{z}\frac{u_{N_{z},k}-u_{N_{z}-1,k}}{h_{z}}+\left(D_{z}\Omega_{z}^{2}+\phi \left(1+\psi \left(u_{N_{z},k}^{2}+v_{N_{z},k}^{2}\right)\right)\right)v_{N_{z},k}\\ -2D_{z}\Omega_{z}\frac{v_{N_{z},k}-v_{N_{z}-1,k}}{h_{z}}-\left(D_{z}\Omega_{z}^{2}+\phi \left(1+\psi \left(u_{N_{z},k}^{2}+v_{N_{z},k}^{2}\right)\right)\right)u_{N_{z},k} \end{array}),\\ k=\bar{1,N_{x}-1},m=\bar{0,N_{t}-1}. \end{array} \end{array}$$ (113)

In the formulae (112), (113) the mesh functions *u*_*j*,*k*_ and *v*_*j*,*k*_ denote the real and imaginary parts of the complex amplitude $A_{j,k}=u_{j,k}+ıv_{j,k},j=\bar{0,N_{z}},k=\bar{0,N_{x}}$. The set of Eqs (110)–(113) is solved by using the Thomas algorithm.

Then, we compute the problem solution near the boundaries along the x-coordinate by using Rosenbrock method with ABCs (90). We denote a number of the grid nodes in the sub-domains along x-coordinate as $N_{x_{R}}$ and write the following finite-difference scheme of the solution computation based on the Rosenbrock method:

$$\begin{array}{c} {\hat{A}}_{Ros}=A+\tau_{R}Re\bar{q},\;k=\bar{0,N_{x_{R}}};\bar{N_{x}-N_{x_{R}},N_{x}},j=\bar{1,N_{z}-1},m=\bar{0,N_{t}-1}, \end{array}$$ (114)

where $Re\bar{q}$, is a real part of the solution for the linear equations

$$\begin{array}{c} \begin{array}{cc} \left(E-\beta \tau_{R}G_{U}\right)\bar{q}=G\left(U\right), & \;k=\bar{1,N_{x_{R}}};\bar{N_{x}-N_{x_{R}},N_{x}-1},\\ \left(E-\beta \tau_{R}G_{U_{b}}\right)\bar{q}=G\left(U_{b}\right), & \;k=0,N_{x},j=\bar{0,N_{z}},m=\bar{0,N_{t}-1}. \end{array} \end{array}$$ (115)

*G*_*U*_ is the Jacobian for the vector *G*(*U*)

$$\begin{array}{c} \begin{array}{c} G(U)=(\begin{array}{c} \frac{D_{z}}{\varepsilon }\Lambda_{\bar{z}z}v_{j,k}+\frac{D_{x}}{\varepsilon }\Lambda_{\bar{x}x}v_{j,k}+\phi \left(1+\frac{\psi }{\varepsilon }\left(u_{j,k}^{2}+v_{j,k}^{2}\right)\right)v_{j,k}\\ -\frac{D_{z}}{\varepsilon }\Lambda_{\bar{z}z}u_{j,k}-\frac{D_{x}}{\varepsilon }\Lambda_{\bar{x}x}u_{j,k}-\phi \left(1+\frac{\psi }{\varepsilon }\left(u_{j,k}^{2}+v_{j,k}^{2}\right)\right)u_{j,k}, \end{array}),\\ k=\bar{1,N_{x_{R}}},\bar{N_{x}-N_{x_{R}},N_{x}-1},j=\bar{1,N_{z}-1},m=\bar{0,N_{t}-1}. \end{array} \end{array}$$ (116)

and $G_{U_{b}}$ is the Jacobian of the vector *G*(*U*_*b*_), which corresponds to the ABCs and is written in the following form

$$\begin{array}{c} \begin{array}{c} G(U_{b})=(\begin{array}{c} 2D_{x}\Omega_{x}\frac{u_{j,1}-u_{j,0}}{h_{x}}+\left(D_{x}\Omega_{x}^{2}+\phi \left(1+\psi \left(u_{j,0}^{2}+v_{j,0}^{2}\right)\right)\right)v_{j,0}\\ 2D_{x}\Omega_{x}\frac{v_{j,1}-v_{j,0}}{h_{x}}-\left(D_{x}\Omega_{x}^{2}+\phi \left(1+\psi \left(u_{j,0}^{2}+v_{j,0}^{2}\right)\right)\right)u_{j,0}\\ -2D_{x}\Omega_{x}\frac{u_{j,N_{x}}-u_{j,N_{x}-1}}{h_{x}}+\left(D_{x}\Omega_{x}^{2}+\phi \left(1+\psi \left(u_{j,N_{x}}^{2}+v_{j,N_{x}}^{2}\right)\right)\right)v_{j,N_{x}}\\ -2D_{x}\Omega_{x}\frac{v_{j,N_{x}}-v_{j,N_{x}-1}}{h_{x}}-\left(D_{x}\Omega_{x}^{2}+\phi \left(1+\psi \left(u_{j,N_{x}}^{2}+v_{j,N_{x}}^{2}\right)\right)\right)u_{j,N_{x}} \end{array}),\\ j=\bar{0,N_{z}},m=\bar{0,N_{t}-1}. \end{array} \end{array}$$ (117)

The set of Eqs (114)–(117) is also solved by using the Thomas algorithm. After that we use two-stage iteration process for solving of the Schrödinger equation in full domain at using CFDS with the Dirichlet BCs.

The first stage of the combined method consists in the problem solution, as it is presented in (99), with the BCs defined by the mesh function ${\hat{A}}_{Ros}$, which is a solution of the Eqs (110) and (111):

$$\begin{array}{c} {\overset{s+1}{{\hat{A}}_{C}}}_{0,k}={\hat{A}}_{Ros\;0,k},\;{\overset{s+1}{{\hat{A}}_{C}}}_{N_{z},k}={\hat{A}}_{Ros\;N_{z},k},\;k=\bar{1,N_{x}-1},m=\bar{0,N_{t}-1}. \end{array}$$ (118)

Here we denote the problem solution on this stage as ${\overset{s+1}{\hat{A}}}_{C}$. It should be emphasized that at the first time layer we use the initial complex amplitude distribution (97).

Thus, we obtain the solution at the first stage of the combined method $\overset{s+1}{\hat{A}}={\overset{s+1}{\hat{A}}}_{C}$.

The second stage of the combined method corresponds to the difference equation solution along x-coordinate. This stage consists in the problem solution, as it presented in formula (101), with BCs defined by the mesh function ${\hat{A}}_{Ros}$, which is a solution of Eqs (114) and (115)

$$\begin{array}{c} {\overset{s+2}{{\hat{A}}_{C}}}_{j,0}={\hat{A}}_{Ros\;j,0},\;{\overset{s+2}{{\hat{A}}_{C}}}_{j,N_{x}}={\hat{A}}_{Ros\;j,N_{x}},\;j=\bar{0,N_{z}},m=\bar{0,N_{t}-1}. \end{array}$$ (119)

Here we denote the problem (96)–(98) solution for this stage as ${\overset{s+2}{\hat{A}}}_{C}$. The mesh function on the upper time layer at zero iteration (*s* = 0) is chosen as it presented in (39) and the condition of iteration process stopping is written in formula (103). Thus, we obtain the solution at the upper time layer.

We should note, that FFT can not be applied instead of the two-stage iteration method because the function values at the corresponding nodes belonging to the boundaries for x and z coordinates do not equal to each other. Moreover, the problem solution under consideration doesn’t possess the symmetry property along z-coordinate.

### The computer simulation results

First, we should note that the 2D problem solution at using the Rosenbrock method leads to significant increasing of the computation time and algorithm complexity because of using the matrix inversion operation. Therefore, we should take enough large mesh steps along the spatial coordinates. However, even at *h*_*z*_ = *h*_*x*_ = 0.1 and the domain *L*_*z*_ = *L*_*x*_ = 10 we need to compute the inverse matrix with dimension of 160,000 at each of time steps. Thus, we can conclude that the Rosenbrock method is unacceptable for the 2D problem solving.

Using of the proposed combined method for the computation of the 2D problem does not require computation of inverse matrices with such big dimension. Thus, the computation time at using the combined method is close to the corresponding computation time at using the CFDS. Nevertheless, usually the computation time using the CFDS is less than the corresponding time at using the combined method. It is due to additional computations using the Rosenbrock method, which occur in combined method. The combined method is effective for a computation in short time interval and in the case of defocusing medium or some other problems, where it is necessary to use more than two iterations for the CFDS. The CFDS gives more advantages for the problems, where control of invariants is required.

Also, one of the advantages of using the combined method is a decrease of the computation cost and allow to write the CFDS for the arbitrary boundary conditions.

## Conclusions

In this paper we have shown the conditional conservatism property of the finite-difference scheme based on the Rosenbrock method for the 1D nonlinear Schrödinger equation or the set of equations. For fixed mesh steps with increasing the computation time interval the difference analogues of the nonlinear Schrödinger equation (or the set of equations) invariants do not preserve. To achieve their conservation as well as corresponding solution accuracy it is necessary to decrease the mesh step of time coordinate. Despite the Rosenbrock method is explicit it requires much more computational time in comparison with corresponding time at using of CFDS because of essential smaller mesh steps which are necessary for the Rosenbrock method. This leads to a loss of the Rosenbrock method’s main advantage at computation during long time interval.

We investigated the effectiveness for the Rosenbrock method for the problem with the ABCs. The results obtained for the linear case and for defocusing medium show that using the finite-difference scheme based on the Rosenbrock method gives the same accuracy order of the problem approximation as the CFDS. For the self-focusing medium it is necessary to decrease mesh step on time coordinate tenfold for the finite-difference scheme based on Rosenbrock method to achieve the accuracy of the solution using CFDS.

To use the advantages of the explicit finite-difference scheme and CFDS we proposed the new combined method for numerical solution of the nonlinear Schrödinger equation (or the set of equations) with ABCs. This method can be more effective than the CFDS under certain conditions. The computer simulation results, obtained using the combined method possess the similar order of accuracy in comparison with the corresponding results, obtained using the CFDS, at appropriate choosing the mesh steps. The combined method possesses also the several advantages over the Rosenbrock method for the 1D nonlinear Schrödinger equation (or set of equations) solution.

Using of the Rosenbrock method for multidimensional case leads to the significant increasing of computation time because of the complexity of the matrix inversion operation. However, it is possible to use the combined method with multistage iterative process. In this case we solve only the 1D difference equation using the Thomas algorithm. Thus, the combined method in the multidimensional case can be quite effective.

Using of the Rosenbrock method is inefficient in practice for solving the 2D problem due to computation of inverse matrices with high dimension. We proposed the combined method, which overcomes this disadvantage. The main advantage of the combined method consists in a possibility of changing of the BCs for the CFDS: instead of using the third type of the BCs one can apply the Dirichlet BCs. The ABCs are used at the stage of using the Rosenbrock method. The advantage of using the combined method appears for the 2D problem solution during short time interval and in the case of laser pulse propagation in defocusing medium. The CFDS is effective for the 2D problem solution during a large time interval and in the case of laser pulse propagation in self-focusing medium.
